# Antimicrobial Functionalized Mesoporous Silica FDU-12 Loaded with Bacitracin

**DOI:** 10.3390/molecules31020340

**Published:** 2026-01-19

**Authors:** Dan Adrian Vasile, Ludmila Motelica, Luiza-Andreea Mîrț, Gabriel Vasilievici, Oana-Maria Memecică, Ovidiu Cristian Oprea, Adrian-Vasile Surdu, Roxana Doina Trușcă, Cristina Chircov, Bogdan Ștefan Vasile, Zeno Dorian Ghizdavet, Denisa Ficai, Ana-Maria Albu, Radu Pericleanu, Andreea Ștefania Dumbravă, Mara-Mădălina Mihai, Irina Gheorghe-Barbu, Anton Ficai

**Affiliations:** 1 Department of Science and Engineering of Oxide Materials and Nanomaterials, Faculty of Chemical Engineering and Biotechnologies, National University of Science and Technology POLITEHNICA Bucharest, Gh. Polizu 1-7, 011061 Bucharest, Romaniavasile.surdu@unitbv.ro (A.-V.S.); cristina.chircov@upb.ro (C.C.);; 2 National Research Center for Food Safety, National University of Science and Technology POLITEHNICA Bucharest, Splaiul Independentei 313, 060042 Bucharest, Romania; ludmila.motelica@upb.ro (L.M.); ovidiu73@yahoo.com (O.C.O.);; 3 National Center for Micro and Nanomaterials, National University of Science and Technology POLITEHNICA Bucharest, Splaiul Independentei 313, 060042 Bucharest, Romania; 4 Advanced Research Center for Innovative Materials, Products and Processes, National University of Science and Technology POLITEHNICA Bucharest, 313 Splaiul Independentei, 060042 Bucharest, Romania; 5 National Institute for Research & Development in Chemistry and Petrochemistry–ICECHIM, Spl. Independentei 202, 060021 Bucharest, Romania; gvasilievici@icechim.ro; 6 Department of Bioresources and Polymer Sciences, Faculty of Chemical Engineering and Biotechnologies, National University of Science and Technology POLITEHNICA Bucharest, Gh. Polizu 1-7, 011061 Bucharest, Romania; 7 Department of Inorganic Chemistry, Physical Chemistry and Electrochemistry, Faculty of Chemical Engineering and Biotechnologies, National University of Science and Technology POLITEHNICA Bucharest, Gh. Polizu 1-7, 011061 Bucharest, Romania; 8 Academy of Romanian Scientists, Ilfov Street 3, 050044 Bucharest, Romania; 9 Department of Materials Science, Faculty of Materials Science and Engineering, Transilvania University of Brasov, 29 Eroilor Blvd., 500036 Brasov, Romania; 10 Department of Botany and Microbiology, Faculty of Biology, University of Bucharest, Intr. Portocalelor No. 1–3, 060101 Bucharest, Romania; 11 Department of Oncologic Dermatology, “Elias” Emergency University Hospital, “Carol Davila” University of Medicine and Pharmacy, 020021 Bucharest, Romania; 12 The Research Institute of the University of Bucharest (ICUB), B.P Hasdeu No. 7, 050095 Bucharest, Romania

**Keywords:** mesoporous silica, poly(N-acryloylmorpholine), bacitracin, drug delivery, vacuum-assisted loading, surface functionalization, skin infection, kinetic model, antimicrobial, anti-biofilm virulence factors modulation, *Staphylococcus* spp.

## Abstract

The threats leading to the extinction of humanity accelerate the evolution and development of materials that are capable of providing conditions for preserving health and, implicitly, life. In our work, we developed drug delivery systems based on mesoporous silica which can deliver an antibiotic, bacitracin, in a more controlled manner. The synthesis of the FDU-12 was performed through a sol–gel method and alternatively functionalized with -NH_2_ groups or with poly(N-acryloylmorpholine) chains. The loading of bacitracin was performed using the vacuum-assisted method we successfully used to load these mesoporous materials preferentially within the pores as proved by the TGA-DSC results. The release was performed in two types of simulated body fluid (SBF) and this process was evaluated with chromatographic method using UV detection. The obtained data were fitted in three mathematical models of kinetic drug release (Weibull model, Korsmeyer–Peppas model, and nonlinear regression). The antimicrobial evaluation demonstrated that bacitracin-loaded FDU-12 formulations exhibited strong activity against both reference and clinical *Staphylococcus* strains. At sub-inhibitory concentrations, all formulations significantly reduced microbial adherence and biofilm formation, although certain strain-dependent stimulatory effects were observed. Furthermore, exposure to sub-MIC levels modulated the production of soluble virulence factors (hemolysins, lipase, and amylase), in a formulation- and strain-dependent manner, underscoring the ability of surface-functionalized FDU-12 carriers to influence bacterial pathogenicity while enhancing antimicrobial efficacy.

## 1. Introduction

Antimicrobial resistance (AMR) is a global challenge that threatens public health and food security, while also being a cause of the slowed progress towards the United Nations proposed sustainable development goals. Various factors, such as improper use of antimicrobial drugs or inadequate sanitation, can help increase the number of cases of AMR and spread this phenomenon [1]. In today’s world, the number of deaths linked to antimicrobial resistance diseases is estimated at 700,000 per year, revealing a potential increase to 10 million in 2050 [2]. The sixth-highest cause for AMR-attributable deaths is represented by bacterial infections of the skin and of subcutaneous tissues [3].

The development and use of nanomaterial-based drug delivery systems can be a key advantage in this fight against microbes. This type of system allows, in addition to the targeted dosing of the drug, the long-term maintenance of an optimal therapeutic concentration in the area of the body affected by disease [4]. Since their discovery [5,6] and their first use as a drug delivery system [7], mesoporous silica micro/nanoparticles are not yet part of many drug formulations approved by authorities, despite their numerous characteristics such as biocompatibility, versatility for functionalization, or high drug-loading capacity [8,9]. There are many examples of mesoporous silica particles loaded with different antibiotics: vancomycin [10], tetracycline [11], cefepime [12], ampicillin [13], amoxicillin [14], aminoglycosides [15], and rifampicin [16].

Due to the presence of silanol groups on the surface, these materials can be functionalized, and this process can improve their properties or provide them with new ones, depending on the applications for which they are intended to be used. The surface of these materials can be functionalized with inorganic particles [17,18], different organic functional groups [19,20], or polymers [21,22], especially to help increase the loading capacity and to improve control of the release process, or they can be functionalized with DNA [23,24], polysaccharides [25], lipids [26,27,28], proteins [29,30], or antibodies [31,32], especially to increase their biocompatibility or to facilitate targeted drug delivery [33].

Among the many functionalization agents that can be used to modify the surface, N-acryloyl-morpholine (NAM) is a disubstituted acrylamide derivative, with an inherently hydrophilic nature and solubility in a wide variety of solvents, from aqueous to organic ones (e.g., chloroform, tetrahydrofuran, 1, 4-dioxane) [34], inducing some specific functionality.

The choice of NAM as a monomer for the modification of silica substrate by graft polymerization is justified, first in terms of its good biocompatibility and secondly by the antigenic effect of its polymers. The presence of pNAM grafts is expected to support and enhance both the biocompatibility and release of the drug loaded on this substrate [35]. Since the pNAM sequence is generated directly on the silica support, on which free radical initiation centers were previously generated, the approached process is of type graft on the surface.

For generating graft initiation centers, a radical redox initiation using cerium sulfate (CS) was preferred, to the detriment of initiating the process with high energy or microwave radiation. This choice is justified by the limitations aimed at degrading the substrate, namely the energy cost. In addition, redox initiation, assuming ambient working temperatures, promotes the growth of polymers on the modified substrate, to the detriment of the development of independent polymerization in the reaction medium [36,37,38,39,40].

The mechanism by which Ce^4+^ ions generate free radicals (initiation centers) on various substrates that possess functional reactive groups (OH; NH_2_) is not generalized. An accepted theory for OH group substrates is the formation of an initiator complex involving OH groups and the Ce^4+^ ion, following the transfer of complex electrons dissociating with the release of Ce^3+^ and the formation of a radical center at the atom adjacent to the OH group [41].

In this paper, we developed three systems based on a type of mesoporous silica with cage structure FDU-12 which were kept unfunctionalized or functionalized with aminopropyl groups or pNAM and loaded with bacitracin. The mesoporous materials with 3D-pore systems are better in terms of facilitating mass diffusion and transportation compared with materials with 1D channels [42], while the choice of FDU-12 was performed based on the larger and more tunable pore diameter, making it implicitly more suitable for loading high molecular compounds such as peptides and small proteins [43], and also considering the intention to develop tunable DDS with shutter-like behavior. Bacitracin is a polypeptidic antibiotic, and was discovered in 1945 during the treatment of a knee injury and isolated from *Bacillus licheniformis* [44]. The mechanism of action is through inhibition of the linear peptidoglycan chains, which are one of the main components of bacterial cell membranes [45]. Due to its nephrotoxicity [46], bacitracin is used often through topical administration, especially against Gram-positive bacteria such as *Staphylococcus aureus*, *Staphylococcus epidermidis*, and *Streptococcus pyogenes* [47,48,49,50]. The interactions between bacitracin and the substrate is particularly influenced due to the surface chemistry of the material as follows: the amino groups modify the surface charge, being positive at physiological pH values, and increase the hydrophilic character due to formation of hydrogen bonds [51,52], while the pNAM is known to increase colloidal stability and to reduce protein adhesion to the surface of the material, since it is highly biocompatible [53].

The study also aimed to evaluate the antimicrobial efficiency, anti-adherence activity, and effects on virulence factor expression of bacitracin and bacitracin-loaded mesoporous silica FDU-12 formulations (FDU-12, FDU-12–NH_2_, and FDU-12pNAM) against both reference and clinical *Staphylococcus* strains. The objective was to elucidate how surface functionalization of the FDU-12 carrier and different bacitracin concentrations influence bacterial growth inhibition, biofilm formation, and the production of soluble virulence factors, thereby assessing the potential of these systems as advanced antimicrobial delivery systems. To ensure a comprehensive assessment of the antimicrobial performance of the bacitracin-loaded FDU-12 systems, both reference and clinical *Staphylococcus* strains were included. Reference strains of *S. aureus* and *S. epidermidis* serve as genetically stable, well-characterized models that allow reproducible measurement of baseline antimicrobial activity and facilitate comparison with previously published data. In contrast, clinical isolates typically exhibit substantially greater phenotypic and genotypic heterogeneity, including altered susceptibility patterns, heightened biofilm-forming ability, and adaptive regulatory shifts shaped by host-associated selective pressures and prior antibiotic exposure. Incorporating clinical *S. aureus* isolates therefore provides a more realistic and clinically relevant representation of the challenges posed by contemporary infections, particularly those involving persistent or device-associated biofilms. By evaluating antimicrobial activity across both reference strains and clinically adapted pathogens, the study strengthens the translational relevance of its findings. This integrated approach ensures that the antimicrobial potential of the FDU-12–based delivery systems is characterized under conditions that more accurately reflect real-world clinical scenarios.

## 2. Results and Discussion

### 2.1. The Determination of Grafting Degree

After performing the calculations, results were obtained as follows: the grafting degree was 32%, the global grafting yield was 7.8%, and the conversion to grafted polymer was 20%. Since the role of polymer grafts is exclusively to protect the antibiotic loaded on silica, the obtained values are satisfactory. The conversion of monomer to graft can also be interpreted as molarities, so one can estimate that the size of the grafts is about 2000 Da, corresponding to a GPn of about 14 NAM.

### 2.2. Zeta Potential Analysis

The zeta potential values of the discharged samples were as follows: for FDU-12, a value of—26.85 mV; for FDU-12-NH_2_, a value of—2.73 mV; and for FDU-12-pNAM, a value of—16.53 mV. The change in potential value demonstrates a compositional change in surface area of FDU-12. There is a drastic reduction in the potential for FDU-12-NH_2_ (24.12 mV) compared to the modified pNAM (10.32 mV) sample. These values are, on the one hand, the consequence of the chemical nature of the graft, but on the other hand, they also represent the degree of grafting, graft–graft interactions, and graft–substrate interactions, respectively. APTES compatibility with the silica substrate favors a higher “load” with aminopropyl sequences, which reduces the surface potential due to the cationic character of the propyl sequence. Thus, the material assembly will function as a proton absorber but, on the other hand, it will favor aggregation with blood sequences and activate the immune system [54,55,56]

A decrease of only 10 mV in potential at pNAM grafting demonstrates a mean grafting density (32%) with oligomeric sequences that do not interact with the substrate due to steric hindrance. Thus it can be said that these grafts function as “sequestrants” which control the respective absorption and desorption processes of the active substance. On the other hand, it shows good colloidal stability [57].

### 2.3. SEM Analysis

**Figure 1:**
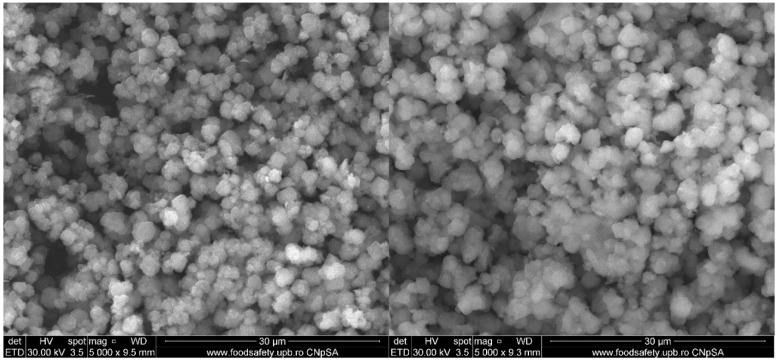
SEM images of FDU-12-pNAM (**left,** unloaded sample; **right,** the loaded one).

The morphology of FDU-12-pNAM is shown in the SEM images (Figure 1). The samples have particles between 1.8 µm and 4.2 µm with a mean diameter of 2.5 µm. After functionalization, the particle size is not modified considerably, and when compared with the pristine FDU-12, the sizes expressed in the literature are quite similar [58]. Instead, the shape of the particles became more irregular and more dispersed, probably due to electrostatic repulsion and steric effects [59,60]. The loading process does not increase significantly the particle size, confirming the fact that the majority of bacitracin is present in the mesopores framework and not on the surface of the support material.

### 2.4. TEM Analysis

**Figure 2:**
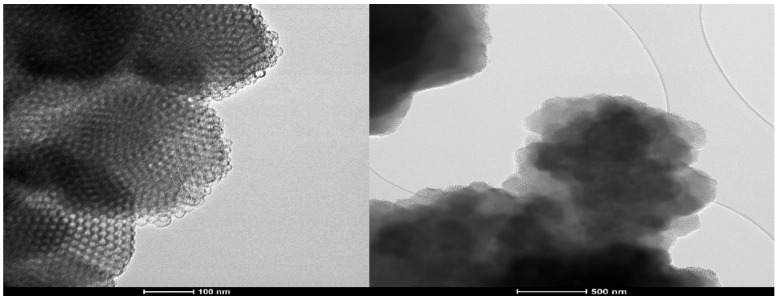
TEM images of FDU-12 sample.

Figure 2 highlights the TEM images of the FDU-12 sample. They reveal the highly porous particles with well-ordered and uniform pores. This type shows a structure with the highest packing density of perfect spheres. Also the formation of stacking faults can be observed in the mesopore framework [61].

### 2.5. N_2_ Absorption/Desorption Analysis

**Figure 3:**
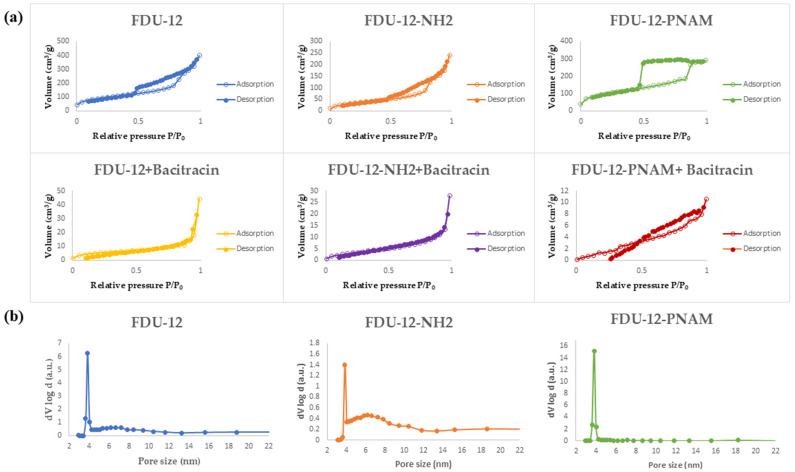
(**a**) N_2_ adsorption/desorption isotherms of the loaded and unloaded samples and (**b**) the pore-size distributions based on BJH method.

The values of parameters such as surface area or pore volume have a major influence in the efficiency of a drug delivery system. A higher surface area is associated often with a higher loading drug capacity and a slower release, while a large pore size can lead to a faster release rate of the drug [62]. According to Figure 3a, all the samples present a type IV hysteresis loop that is specific for mesoporous materials [63]. Certainly, the loading of the mesoporous structures with bacitracin lead to a major modification of the absorption–desorption isotherms with N_2_ and these hysteresis loops are not so visible because of the partial loading of the pores with bacitracin.

The pore-size distributions obtained through the BJH method applied to the adsorption branch of the isotherms (Figure 3b) reveal, for all samples, a high peak at a value of ~4 nm, meaning that this pore size is representative of all three supports, which means that the modification is mainly occurring on the surface of the particles and not inside the pores.

Table 1 summarizes the surface area and pore volume determined from BET analysis. It can be seen that the aminopropyl-functionalization process with APTES led to a considerable decrease in the specific surface area, with the explanation being that the aminopropyl groups are not only attached on the surface of the material but also inside the pores, which is comparable with the process of functionalization with the pNAM chains, which are mainly on the surface of the material. For the loaded samples, these parameters have much lower values, highlighting the presence of bacitracin inside the pores.

### 2.6. XRD Analysis

**Figure 4:**
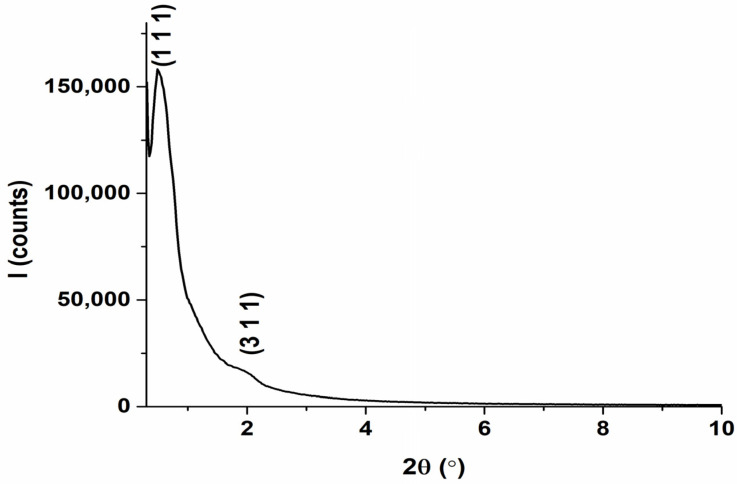
Small-angle diffractogram for FDU-12.

Figure 4 shows the SAXS pattern of the calcined sample FDU-12. The diffractogram reveals peaks assigned to (110) and (311) Miller indices. These peaks are correlated with the diffraction pattern of a face-centered cubic (fcc) structure, with a space group of Fm-3m. The broadening of peaks can be due to polydispersity, disorder, or crystal defects, which can also contribute to this phenomenon [64,65,66].

### 2.7. FT-IR Analysis

**Figure 5:**
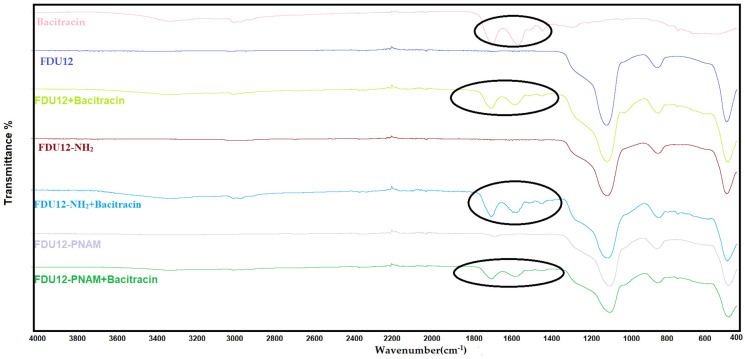
Infrared spectra of samples before and after loading of bacitracin (oval areas indicate the spectral regions that prove the presence of the bacitracin loaded into the mesoporous silica).

The FT-IR data (Figure 5) show specific mesoporous silica peaks for unloaded samples: the asymmetric stretching vibration of Si-O-Si are approximatively at 1060 cm^−1^ and from the same group the symmetric stretching vibrations are at 800 cm^−1^. At 440 cm^−1,^ the peak associated with the stretching vibration of the Si-O bond can be seen [67]. The functionalized unloaded samples present specific functional group peaks: for the aminopropyl-functionalized sample, the peak occurs around 3200 cm^−1^, and the peak corresponding to –NH_2_ stretching is present with low intensity due to the low degree of functionalization, while the polymer-functionalized sample at 1629 cm^−1^ represents the peak of the C=O stretching vibration of amide group. For the loaded samples the specific peaks belonging to bacitracin can be identified, if we overlap the spectra of pure bacitracin and the spectra of the loaded samples. Can be observed similar peaks confirming the presence of bacitracin, especially in the region 1380 cm^−1^–1800 cm^−1^ (marked in the Figure 5 with a black circle). Also, outside this region, there is a peak at the 3300 cm^−1^ band resulting from the -NH/-OH stretching vibrations [68]. The specific peaks (Table 2) of the bacitracin are slightly shifted, proving that there are interactions developing between the support and the antibiotic.

### 2.8. TG-DSC Analysis

**Figure 6:**
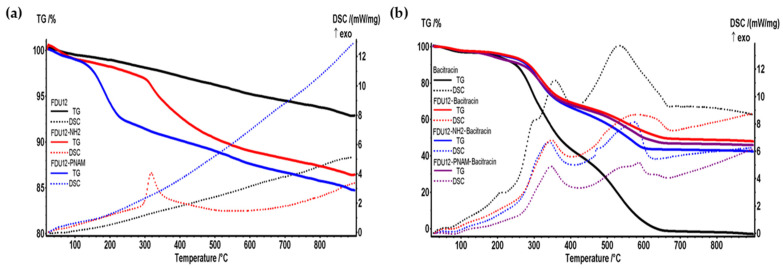
(**a**) TGA-DSC curves of unloaded samples (unfunctionalized FDU-12, the aminopropyl-functionalized one and the sample with the pNAM grafted). (**b**) The thermal analysis for the bacitracin and bacitracin-loaded samples.

The efficiency of functionalization processes and the efficiency of loading with bacitracin in materials can both be evaluated with the thermal analysis TGA-DSC. In the first case the blank is the unfunctionalized sample and in the second one it is the unloaded sample. As can be seen in Figure 6a, after the aminopropyl-functionalization process the mass loss of sample achieves an addition of 6.95% and after the grafting of polymer the mass loss is increased by 8.8%.

For FDU-12 and FDU-12-NH_2_ the mass loss recorded in the range RT-120 °C was low because it represents the amount of weakly bounded water, as well as physically adsorbed molecules from the surface and inside the pores [69]. After 120 °C the water bounded into the pores of silica is lost, together with condensation of -OH moieties and the process of densification of silica takes place. The pNAM organic part is degraded mostly at the temperature interval 120–250 °C, when a mass loss of 6.52% is recorded. The lack of a clear thermal effect indicates that the process is a mix of decomposition, fragmentation, and oxidation of the smaller molecules. In the interval 250–440 °C, the organic part that is introduced in the FDU-12–NH_2_ functionalization process is oxidized, and the process is accompanied by an exothermic effect with maximum value at 318.1 °C (Figure 6a). The table below (Table 3) presents the principal data from the thermal analysis for unfunctionalized and functionalized FDU-12 samples.

Thermal analyses for the loaded samples are similar due to the dominance of the degradation pathway of bacitracin that represents about half of the samples’ mass (Figure 6b). Up to 120 °C, the mass loss is ~3%, representing elimination of the residual solvent molecules. The degradation of the organics starts after 250 °C, in a series of degradative–oxidative processes, as indicated by the multiple exothermic peaks from 337 to 349 °C and 574–587 °C (for the loaded samples). These exothermic effects appear shifted when compared with the thermal analysis of the bacitracin; additionally, the effects from 209 and 299 °C are missing in the loaded samples. This confirms the successful loading process and indicates possible bonds between bacitracin and the FDU support.

After 440 °C, the residual carbonaceous mass is burned away when a second high mass loss is recorded (Table 3).

The incorporation of bacitracin through the vacuum-assisted method in all samples was very efficient. The aminopropyl-functionalized sample has a higher loading content, perhaps due to the formation of a higher number of hydrogen bonds between bacitracin and the support.

### 2.9. HPLC-DAD Release Evaluation

**Figure 7:**
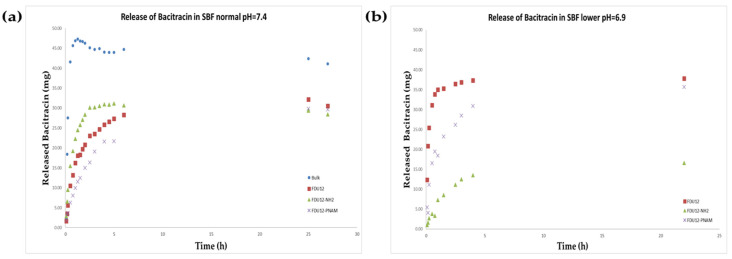
Release of bacitracin in SBF medium at (**a**) normal pH (7.4) and (**b**) lower pH (6.9).

The release profile of bacitracin in SBF is presented in Figure 7a. It can be observed that the drug delivery systems induce an extended release of bacitracin over time. The maximum recovery regarding the bulk sample (95.32%) occurred in the first 75 min, and for FDU-12-NH_2,_ the maximum (59.66%) is reached at 5 h, while for pristine FDU-12 (65.49%) and for the one with pNAM grafting, the maximum of 64.52% is reached over 24 h.

Regarding the release behavior in the SBF with lower pH (pH = 6.9, Figure 7b), the release profile is different. All samples reach the maximum of released bacitracin at approximately the same timepoint, over 22 h. The aminopropyl-functionalized sample has the lowest release content of 32.94%, the unfunctionalized sample has a maximum of 75.75%, and the pNAM-grafted sample has a maximum release of 75.11%. Bacitracin being an amphiphilic peptide when is in a medium with a lower pH, it has more protonated groups. Regarding the contribution of the support, J.M. Rosenholm et al. demonstrate the fact that on the surface, they consist of approximatively 20% silanol groups with a pK_a_ < 2 (isolated silanol groups), while the remaining 80% have a pK_a_>8 (geminal and vicinal silanol groups) [70]. So, in the case of the unfunctionalized FDU-12, there are more negative charges on the surface at a higher pH, and implicitly more electrostatic interactions with bacitracin. For FDU-12-NH_2,_ the release is delayed in the medium with the lower pH because the amino groups of the support are more polarized and act as a gate based on electrostatic repulsion, which keeps the bacitracin inside the pores and thus slows down the release process. It is known that the tertiary amino groups from poly(N-acryloylmorpholine) free molecule in aqueous solution have a pK_a_ of around 8.3 [71], so it is expected to be protonated and, like in the case of FDU-12-NH_2,_ to have a role in delaying bacitracin release. But for the pNAM chains being grafted, these values of pK_a_ can be influenced by electrostatic interactions with the surface or by ionic strength and composition of SBF medium. In comparison with the release in SBF at normal physiological pH (7.4), the samples FDU-12 and FDU-12-pNAM have an enhanced release process in the medium with lower pH. Also, based on our measurements, even after 74 h, the concentration of released bacitracin continues to be high (70.28% for FDU-12 and 70.54% for FDU-12-pNAM).

### 2.10. Kinetic Models

Data fitting for W and K-P models led to the values of (a, b) and (k, n) given in Table 4. In the same Table, R^2^ values prove that both models performed well (R^2^ ≥ 0.94), noticing that W model covers 100% of the original datasets rather than only 60%, as in the case of the K-P model. When tested for all datasets, the K-P model recorded a decrease in efficiency (0.66 ≤ R^2^ ≤ 0.94).

Logarithmic regression (LnR) generally performed better in the case of 60% data fitting (0.85 ≤ R^2^ ≤ 0.99), as in the case of 100% (0.56 ≤ R^2^ ≤ 0.96).

Models’ parameters and R^2^ values of the three models used are shown in Table 4, describing two cases: (i) pH = 7.4 and (ii) pH = 6.9. From the analysis of R^2^ values, it could be found that W model is the best one, even though it was fitted with the entire database.

**Figure 8:**
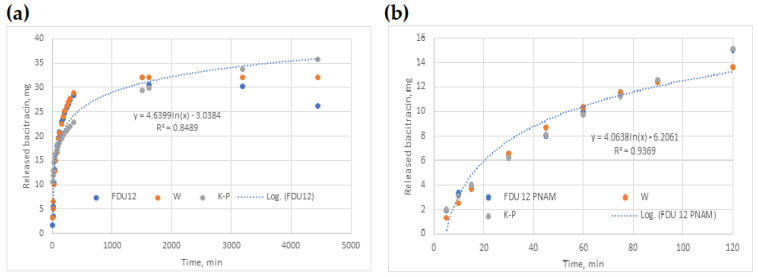
Release curve fitted by the three models in the case of (**a**) FDU-12, for 100% datasets used and pH 7.4; (**b**) FDU-12-pNAM, for 60% datasets used and pH 7.4.

When the three models were fitted for 100% of the datasets, the W model was the closest one to the experimental curve, as shown in Figure 8a. Also, LnR and K-P tend to provide closer results.

Figure 8b shows the case when all three models were fitted for 60% of the datasets, with W and K-P being both close to the experimental points.

### 2.11. Antimicrobial Activity of Bacitracin and FDU-12/FDU-12-NH_2_/FDU-12-pNAM Loaded with Bacitracin Against Reference and Clinical Gram-Positive Strains

#### 2.11.1. Qualitative Screening of the Antimicrobial Activity of FDU-12/FDU-12-NH_2_/FDU-12-pNAM Loaded with Bacitracin Against Reference and Clinical Gram-Positive Strains

**Figure 9:**
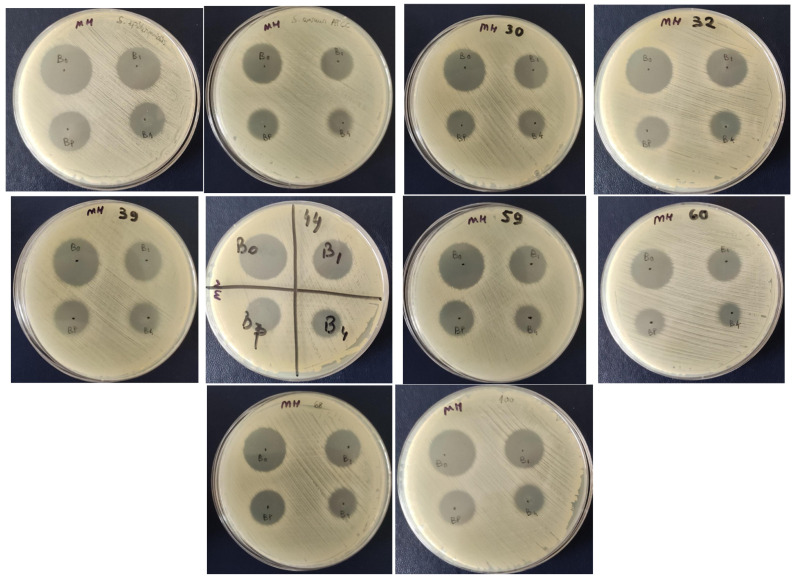
Qualitative screening of the antimicrobial activity Bacitracin and Bacitracin–FDU-12 derivatives against reference and clinical *S. aureus* strains.

The qualitative evaluation of the antimicrobial activity of bacitracin and FDU-12/FDU-12-NH_2_/FDU-12-pNAM loaded with bacitracin (B0 = bacitracin; B1 = FDU-12 + Bacitracin; B4 = FDU-12-NH_2_ + Bacitracin; BP = FDU-12-pNAM + Bacitracin) against reference and clinical Gram-positive strains belonging to *S. aureus*, demonstrated the efficiency in the following decreasing order, revealed by the inhibition zone diameters: B0 [*S. epidermidis* ATCC 12228 *= S. aureus*_30_ = *S. aureus*_32_ = *S. aureus*_39_ = *S. aureus*_44_ = *S. aureus*_59_ = *S. aureus*_68_ = *S. aureus*_100_ (AU = 3) > *S. aureus* ATCC 25923 = *S. aureus*_60_ (AU = 2)]; B1 [*S. epidermidis* ATCC 12228 (AU = 3) > *S. aureus* ATCC 25923 = *S. aureus*_30_ = *S. aureus*_32_ = *S. aureus*_39_ = *S. aureus*_44_ = *S. aureus*_59_ = *S. aureus*_60_ = *S. aureus*_68_ = *S. aureus*_100_ (AU = 2)]; BP [*S. epidermidis* ATCC 12228 = *S. aureus* ATCC 25923 = *S. aureus*_30_ = *S. aureus*_32_ = *S. aureus*_39_ = *S. aureus*_44_ = *S. aureus*_59_ = *S. aureus*_60_ = *S. aureus*_68_ = *S. aureus*_100_ (AU = 2)] and B4 (*S. epidermidis* ATCC 12228 = *S. aureus* ATCC 25923 = *S. aureus*_30_ = *S. aureus*_32_ = *S. aureus*_39_ = *S. aureus*_44_ = *S. aureus*_59_ = *S. aureus*_68_ = *S. aureus*_100_ (AU = 2) > *S. aureus*_60_ (AU = 1)] (Table 5, Figure 9).

#### 2.11.2. Quantitative Evaluation of the Antimicrobial Activity of Bacitracin and Bacitracin–FDU-12 Derivatives

**Figure 10:**
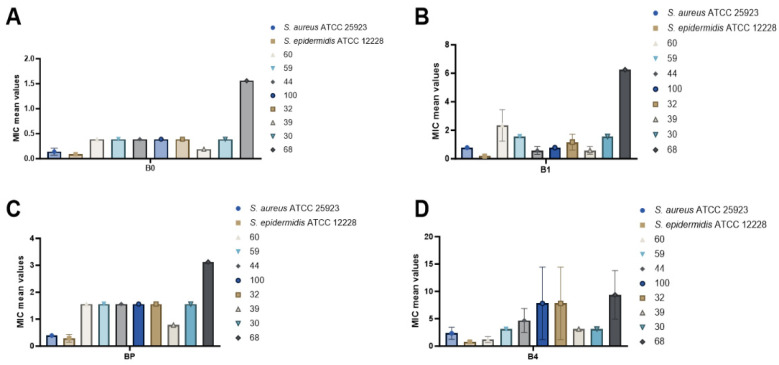
Average MIC values for the tested solutions ((**A**)—B0; (**B**)—B1; (**C**)—BP and (**D**)—B4) against *S. aureus* ATCC 25923, *S. epidermidis* ATCC 12228, and clinical *S. aureus* isolates.

Following the quantitative evaluation of the antimicrobial activity of Bacitracin and Bacitracin–FDU-12 derivatives against both reference and clinical *S. aureus* strains, the highest efficiency was observed for the reference strain *S. epidermidis* ATCC 12228, with the MIC values ranked as follows: B0 (0.09 mg/mL) > B1 (0.19 mg/mL) > BP (0.29 mg/mL) > B4 (0.78 mg/mL). This was followed by *S. aureus* ATCC 25923, showing a similar trend: B0 (0.14 mg/mL) > BP (0.39 mg/mL) > B1 (0.78 mg/mL) > B4 (2.34 mg/mL) (Table 6, Figure 10).

**Figure 11:**
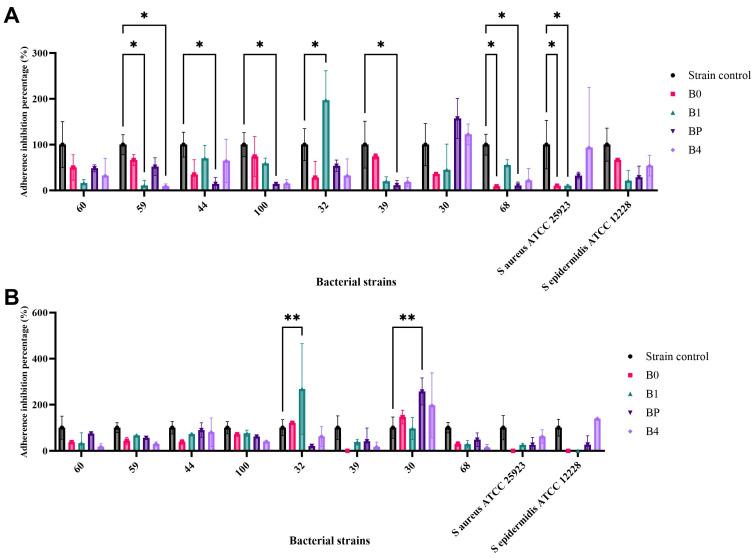
Percentage of adherence inhibition (%MAI) for the tested Bacitracin and Bacitracin–FDU-12 formulations at sub-inhibitory concentration ((**A**) MIC/2 and (**B**) MIC/4) against *S. aureus* ATCC 25923, *S. epidermidis* ATCC 12228, and clinical *S. aureus* isolates. Statistical significance was determined using Dunnett’s multiple comparisons test (* *p*< 0.05, ** *p*< 0.01).

For clinical *S. aureus* isolates, the antimicrobial efficiency decreased in the following order: B0 > B1 > BP > B4 (Table 6, Figure 10). When comparing the activity of each formulation across the tested strains, patterns were observed as follows:

- B0: *S. epidermidis* ATCC 12228 (0.09 mg/mL) > *S. aureus* ATCC 25923 (0.14 mg/mL) > clinical *S. aureus* strain 39 (0.19 mg/mL).
- B1: *S. epidermidis* ATCC 12228 (0.19 mg/mL) > *S. aureus* strain 39 = strain 44 (0.58 mg/mL).
- BP: *S. epidermidis* ATCC 12228 (0.29 mg/mL) > *S. aureus* ATCC 25923 (0.39 mg/mL) > *S. aureus* strain 39 (0.78 mg/mL).
- B4: *S. epidermidis* ATCC 12228 (0.78 mg/mL) > *S. aureus* strain 60 (1.17 mg/mL) (Table 6, Figure 11).
- Notably, three of the tested formulations, B0 (pure Bacitracin), B1 (FDU-12 + Bacitracin), and BP (FDU-12-pNAM + Bacitracin), exhibited significant antimicrobial activity against the clinical *S. aureus* strain 39 (Table 6, Figure 10).

The reduced susceptibility of clinical *S. aureus* isolates to bacitracin and its derivatives is likely attributable to a combination of genetic, physiological, and ecological adaptations that arise during infection and clinical antibiotic exposure. Unlike reference strains, clinical isolates typically display substantial genomic heterogeneity, including point mutations, gene amplifications, and acquisition of mobile genetic elements (e.g., plasmids, transposons, SCCmec elements) that encode resistance determinants and stress–response functions [72,73]. These features emerge under sustained selective pressures in vivo, where bacterial populations must withstand host immune defenses, nutrient limitation, oxidative stress, and repeated or sub-optimal antibiotic treatments [74]. Furthermore, clinical isolates frequently exhibit an enhanced capacity for biofilm formation on host tissues or indwelling medical devices. The resultant extracellular polymeric matrix impedes antimicrobial diffusion, alters local microenvironmental conditions (e.g., pH, oxygen tension), and supports the formation of metabolically inactive persister cells, collectively reducing the efficacy of bacitracin-based formulations and contributing to persistent infection phenotypes [73,75]. In addition, strain-specific variation in the activity of global regulatory systems such as *agr*, *sarA*, and *sigB* modulates the expression of virulence determinants, cell-envelope modifying enzymes, and stress–response pathways, thereby influencing both antimicrobial susceptibility and adaptive survival strategies relative to reference strains [76,77].

Several studies have reported variable susceptibility of *S. aureus* to bacitracin, with MIC values ranging from 16 to 64 µg/mL for susceptible isolates and higher for clinical or multidrug-resistant strains [78]. This pattern is consistent with the higher MIC values observed in the present study, suggesting decreased susceptibility among clinical isolates. The higher numerical MICs can be attributed to the fact that values were expressed per total formulation mass (mg/mL) rather than per active bacitracin content, reflecting the well-known “mass-of-carrier” effect in mesoporous silica nanoparticle-based antibiotic systems. As highlighted in previous studies, functionalized mesoporous silica nanoparticle maintain or only slightly modify the intrinsic antimicrobial activity of the incorporated drug unless specifically designed for controlled release or synergistic delivery [79].

#### 2.11.3. The Impact of Bacitracin and Bacitracin–FDU-12 Derivatives on Biofilm Formation by *S. aureus* ATCC 25923, *S. epidermidis* ATCC 12228, and Clinical *S. aureus* Isolates

At sub-inhibitory concentrations corresponding to MIC/2, all four tested formulations exhibited a reduction in biofilm formation relative to the untreated control (100%). Specifically, significant decreases in biofilm formation ability were observed as follows. B0: *S. aureus* strain 68 (91%, *p* = 0.032) and *S. aureus* ATCC 25923 (89.7%, *p* = 0.036); B1: *S. aureus* ATCC 25923 (89.8%, *p* = 0.036) and *S. aureus* strain 59 (89.4%, *p* = 0.037); BP: *S. aureus* strain 68 (89.5%, *p* = 0.036), strain 39 (88.3%, *p* = 0.040), and strain 100 (85.9%, *p* = 0.047); B4: *S. aureus* strain 59 (90.9%, *p* = 0.033). These findings indicate a marked inhibitory effect on biofilm formation at relatively low (sub-MIC) concentrations, suggesting potent anti-biofilm activity of the tested compounds. In contrast, B1 treatment resulted in a statistically significant increase in biofilm formation for *S. aureus* strain 32, with an MAI value of 97.19% (*p* = 0.020) compared to the untreated control, indicating a strain-dependent response (Table 7, Figure 11A).

At sub-inhibitory concentrations corresponding to MIC/4, the impact of B1 (FDU-12 + Bacitracin), and BP (FDU-12-pNAM + Bacitracin) on biofilm formation by *S. aureus* strain 32 and *S. aureus* strain 30, respectively, showed a statistically significant increase in bacterial adherence to the inert substratum, with an MAI% of 268.15% (*p* = 0.0016) and 257.35 (*p* = 0.0032) relative to the strain control (Table 8, Figure 11B).

The obtained results showed that at MIC/2, all formulations reduced microbial adherence and biofilm formation in several *S. aureus* strains (approximately 86–91% inhibition). However, at MIC/4, a strain-dependent increase in biofilm formation was observed (B1 in *S. aureus* strain 32 and BP in *S. aureus* strain 30), indicating that lower sub-inhibitory concentrations may stimulate biofilm development. This outcome is consistent with other studies reporting that sub-inhibitory levels of different antibiotics can either suppress or induce biofilm formation in *S. aureus*, depending on the antibiotic type, concentration, and genetic background of the strain. Such findings underscore the dual nature of sub-inhibitory antibiotic exposure and reinforce the importance of avoiding under-dosing, particularly when using carrier-based formulations [80]. Therefore, anti-biofilm efficiency should be validated across a range of sub-MICs to accurately assess formulation performance and minimize the risk of biofilm stimulation.

Evaluation of Bacitracin and Bacitracin–FDU-12 derivatives at sub-inhibitory concentrations (MIC/2 and MIC/4) revealed pronounced differences in biofilm-associated responses between reference and clinical *S. aureus* isolates. While all formulations produced consistent biofilm inhibition at MIC/2, several clinical isolates displayed concentration-dependent and strain-specific increases in adherence at MIC/4. This paradoxical enhancement of biofilm formation at lower antimicrobial exposure levels is characteristic of clinical isolates with highly plastic or deregulated biofilm regulatory systems. Sub-inhibitory antimicrobial concentrations can act as environmental cues that activate stress–responsive pathways, downregulate quorum-sensing systems (particularly *agr*), or upregulate adhesion encoding genes and polysaccharide intercellular adhesin synthesis, thereby promoting compensatory biofilm production. These divergent behaviors highlight fundamental differences in regulatory circuit dynamics between reference strains and clinical isolates, underscoring the complexity of bacterial adaptation under sub-inhibitory antibiotic stress.

#### 2.11.4. The Influence of Bacitracin and Bacitracin–FDU-12 Formulations at Sub-Inhibitory Concentration (MIC/2 and MIC/4) on the Ability of Bacterial Strains to Secrete Soluble Virulence Factors

##### Lecithinase Production

The effect of Bacitracin and Bacitracin–FDU-12 formulations on lecithinase activity was assessed across six bacterial strains.

For *S. aureus* ATCC 25923, lecithinase production remained largely unchanged following treatment, with no statistically significant differences compared to the untreated control. Nonetheless, a moderate inhibitory effect was detected in the presence of Bacitracin (B0) at sub-inhibitory concentrations (MIC/2 and MIC/4), resulting in 25% and 12.5% reduction in enzyme activity, respectively, suggesting a strain-dependent response (Supplementary Table S2, Table 9). In contrast, exposure to the composite formulations B1 and BP led to a stimulatory effect on lecithinase production, with the most pronounced increase (37.5%) observed at the MIC/2 concentration.

For the *S. aureus* clinical isolates, no statistically significant differences in lecithinase production were observed compared to the untreated control. In *S. aureus* strain 68, inhibitory effects were detected in the presence of B0 at MIC/4 (25%), B4 at MIC/2 (50%), and BP at MIC/2 (25%) (Supplementary Table S3, Table 9). In *S. aureus* strain 32, a moderate inhibition was observed with BP at MIC/2 and MIC/4 (12.5% and 25%, respectively) (Supplementary Table S4, Table 9). The remaining *S. aureus* clinical strains exhibited a dose-dependent response. For instance, in *S. aureus* strain 100, modest inhibition was recorded with B0 (MIC/4), B1 (MIC/2), and BP (MIC/2) treatments (22.3%). Conversely, in *S. aureus* strains 59 and 60, lecithinase secretion was stimulated in the presence of B1 at MIC/2 and B4 at MIC/4, showing up to 28.5% enhancement compared to the control (Supplementary Table S5, Table 9).

##### Caseinase Production

The effect of Bacitracin and Bacitracin–FDU-12 formulations at sub-inhibitory concentrations on caseinase activity was evaluated in ten bacterial strains. In *S. aureus* ATCC 25923, caseinase production remained largely unaffected by treatment, with no statistically significant differences relative to the untreated control (Supplementary Table S6, Table 10). Among the clinical isolates, the response varied depending on the formulation and concentration applied. For instance, stimulation of caseinase secretion was observed in *S. aureus* strain 39 following exposure to B0 at MIC/2 and BP at MIC/4 (57.15%, *p* = 0.0036), as well as in *S. aureus* strain 59 with B1 at MIC/2 and BP at both sub-inhibitory concentrations (50%, *p* = 0.0144) and in *S. aureus* strain 100 with B1 at MIC/4 (50%, *p* = 0.0144). Conversely, inhibition of caseinase activity was detected under treatment with B1 at MIC/4, BP at MIC/4, and B4 at MIC/2, resulting in 35.8%, 42.9%, and 42.9% reduction, respectively (*p* = 0.0489) (Supplementary Table S6, Table 10), in the case of *S. aureus* strain 44. For the remaining strains, no statistically significant differences in enzyme production were noted compared to the control (Supplementary Tables S7 and S8, Table 10).

##### Amylase Secretion

The impact of Bacitracin and Bacitracin–FDU-12 formulations at sub-inhibitory concentrations on amylase activity was assessed in ten bacterial strains. In the reference strains, amylase secretion remained largely unaffected by treatment, with no statistically significant differences compared to the untreated control (Supplementary Table S10, Table 11). The highest inhibition of enzyme production was observed in two clinical isolates, *S. aureus* 68 and *S. aureus* 100, following exposure to B1 at MIC/2, and to BP and B4 at both sub-inhibitory concentrations, resulting in a 50% reduction in activity (*p* ≤ 0.0001) (Supplementary Table S9, Table 11). Similarly, *S. aureus* 59 exhibited marked inhibition of amylase secretion when treated with B0, B1, and BP at MIC/2 and MIC/4 (50%, *p* = 0.0024). In contrast, *S. aureus* strain 39 demonstrated enhanced amylase production in the presence of B1 at MIC/4, with a 66.7% increase relative to the control (*p* = <0.0001) (Supplementary Table S10, Table 11).

##### Lipase Secretion

The effect of Bacitracin and Bacitracin–FDU-12 formulations at sub-inhibitory concentrations on lipase activity was evaluated in nine bacterial strains. The strongest inhibitory response was recorded for *S. epidermidis* ATCC 12228, showing a 77% reduction in lipase production after treatment with B4 at MIC/2 (*p* = < 0.0001), followed by B0 at MIC/4 (62%, *p* = 0.0018), B0 at MIC/2 (54%, *p* = 0.0082), and B4 at MIC/4 (54%, *p* = 0.0082) (Supplementary Table S11, Table 12). In contrast, several *S. aureus* clinical isolates exhibited stimulation of lipase secretion under specific treatment conditions; for instance, exposure to B0 at MIC/2 and BP at MIC/4 in *S. aureus* strain 39 (57%, *p =* 0.0044); B1 at MIC/2 and BP at both sub-inhibitory concentrations in *S. aureus* strain 59 (50%, *p* = 0.0165); and B1 at MIC/4 in *S. aureus* strain 100 (50%, *p* = 0.0165) (Supplementary Table S11, Table 12). For *S. aureus* strain 68, lipase activity remained comparable to that of the untreated control, with no statistically significant differences detected (Supplementary Table S12, Table 12).

##### Hemolysin Production

The effect of Bacitracin and Bacitracin–FDU-12 formulations at sub-inhibitory concentrations on hemolysin production was evaluated in nine *S. aureus* strains. A significant reduction in hemolysin synthesis was observed in several strains, indicating inhibition of virulence factor expression. The strongest inhibitory response was recorded for *S. aureus* strain 100 treated with Bacitracin (B0) at MIC/2 and MIC/4, showing a 79% decrease compared to the untreated control (*p* < 0.0001). This was followed by *S. aureus* strain 30 (B1, MIC/2 and MIC/4; 77% inhibition, *p* < 0.0001), *S. aureus* strain 32 (B1, MIC/2 and MIC/4; 75% inhibition, *p* < 0.0001), and *S. aureus* strain 59 (B4, MIC/4; 50% inhibition, *p* < 0.0001) (Supplementary Table S13, Table 13).

Conversely, a statistically significant increase in hemolysin production was detected in two clinical isolates: *S. aureus* strain 60 exposed to B0 at MIC/2 (157% of control, *p* < 0.0001) and *S. aureus* strain 39 exposed to B1 at MIC/4 (150% of control, *p* < 0.0001) (Supplementary Table S13, Table 13).

In the present study, sub-inhibitory exposure to the tested formulations produced strain-specific alterations in the expression of soluble virulence factors, including hemolysins, lipase, amylase, caseinase, and lecithinase, ranging from pronounced inhibition to measurable stimulation. Such variability likely reflects differential engagement of stress–response systems and transcriptional regulators that coordinate virulence expression in *S. aureus*. Overall, these findings illustrate that the antibacterial formulations interact with clinical isolates in multifaceted ways that extend beyond growth inhibition, affecting biofilm development, virulence regulation, and adaptive physiological states.

These findings are consistent with previous data demonstrating that antibiotics at sub-inhibitory concentrations can modulate *S. aureus* virulence through global regulatory systems such as *agr* and *sarA*, as well as through stress–response pathways. Notably, cell wall active agents often enhance the production of different exotoxins, whereas ribosome-targeting antibiotics tend to suppress them [81]. Our results extend this paradigm by showing that carrier functionalization specifically, -NH_2_, pNAM, or unmodified FDU-12 surfaces can further influence both the direction and magnitude of virulence modulation, likely due to differences in local drug release kinetics and surface cell interactions. This observation highlights an underexplored aspect of mesoporous silica nanoparticle systems. Recent studies have emphasized that rational surface engineering of mesoporous silica nanoparticles can be strategically employed to modulate bacterial adherence, biofilm formation, and the modulation of virulence-associated phenotypes, thereby supporting the rationale for the functionalization strategies implemented in this study [82].

## 3. Materials and Methods

### 3.1. Materials

All reagents in this study were used without further purification. Triblock co-polymer Pluronic F127, trimethylbenzene (TMB), tetraethylortosilicate (TEOS), aminopropyl-triethoxysilane (APTES), KCl, NaCl, MgCl_2_۰6H_2_O, CaCl_2_, trishidroxiethylaminomethan, toluene, bacitracin, HCl, H_2_SO_4_, NaHCO_3_, N-acryloylmorpholine, CeSO_4_ were obtained from Sigma-Aldrich Merck (Dartmand, Germany).

### 3.2. Methods

#### 3.2.1. Mesoporous Silica Nanoparticles FDU-12 Synthesis

**Figure 12:**
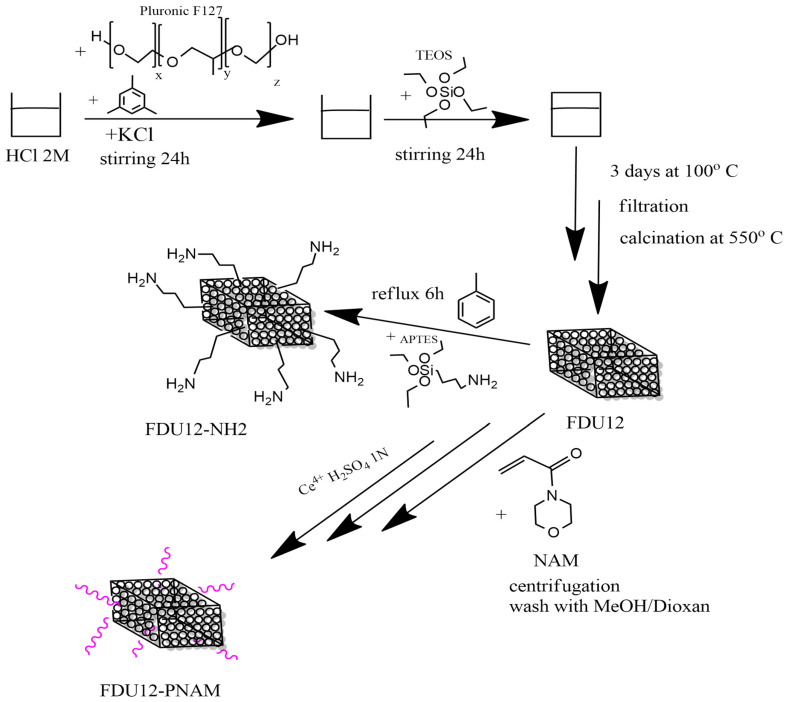
Schematic synthesis of samples.

The synthesis of the FDU-12 mesoporous silica nanoparticles was performed according to the method developed by Yu et al. [83] and based on a sol–gel method using Pluronic F127 as soft template and TMB as a swelling agent dissolved with KCl in a solution of HCl 2 M. After 24 h of stirring the silica source, TEOS is added and is left to mix for another 24 h. The next step is to put the sample at thermal treatment for 3 days at 100 °C, then it is filtered and left to dry at ambient temperature. To remove the organic template, the material is calcinated for 6 h at 550 °C. The schematic preparation procedure is presented in Figure 12.

#### 3.2.2. Functionalization of FDU-12 with Aminopropyl Groups

For aminopropyl-functionalization, the FDU-12 sample is first dried in a vacuum oven for 1 h at 40 °C. After that, the sample is immersed in toluene where APTES is added and it is maintained for a minimum of 6 h at reflux [84]. For better efficiency of the functionalization process, the quantity of APTES is optimized based on the estimation of the number of hydroxyl groups from the surface of FDU-12 [85].

#### 3.2.3. Functionalization of FDU-12 with pNAM

The modification of the FDU-12 substrate with NAM started from a substrate composition that was calculated based on the predetermined structural-dimensional characteristics of silica: average content of OH groups of 3.02 × 10^−3^ mmol OH/g. So, n_Ce4+_/mol OH = 10^−3^; n_NAM_ = n_OH_; [M]_0_ = 3 mol/L. As a source of Ce^4+^, a solution of Ce(SO_4_)_2_ in H_2_SO_4_ 1N (3 × 10^−4^ mol/L) was used as solvent dioxane.

The synthesis procedure is as follows: dispersion of mesoporous silica FDU-12 in the solvent at room temperature (about 15 min at 700 rot/min); addition of the initiator solution, keeping the same conditions for about 15 min; addition of monomer, conditioning by nitrogen purging; stabilization of temperatures at 35 °C with the same stirring regime; and maintenance of the reaction mass under these conditions for 6 h. When stopping the process, the cooled reaction mass is centrifuged (30,000 rot/min), separated, and washed with CH_3_OH/Dioxan mixture (1:9 vol) until colorless and dried at 40 °C for 24 h.

#### 3.2.4. The Determination of Grafting Degree

The grafting degree, obtained after grafting, is calculated using relation Equation (1):

(1) $$\%\;grafting=\frac{m_{modified\;silica}-m_{silica}}{m_{silica}}\times 100$$

where $m_{silica}$ is the initial mass of silica used in reaction (g); $m_{modified\;silica}$ is the mass of grafted silica after washing and drying (g).

The global grafting yield was calculated using relation Equation (2):

(2) $$global\;yield=\frac{m_{modified\;silica}}{m_{modified\;silica}+m_{monomer}}\times 100\;$$

where $m_{monomers}$ is the initial mass of monomer(s) introduced in the grafting reaction (g).

The conversion of the monomer to grafted polymer was calculated using Equation (3):

(3) $$conversion\;to\;grafted\;polymer=\frac{m_{modified\;silica-m_{silica}}}{m_{monomer}}\times 100\;$$

#### 3.2.5. Loading of Bacitracin in the Pores of the Samples

Due to its high solubility in water, the loading of the bacitracin was performed using the vacuum-assisted method developed by our group, from aqueous solution [10,69,85]. The first step is devoted to the removal of water and air from pores of the FDU-12 mesoporous material (1 g) and was achieved by vacuum drying at room temperature with a vacuum rate of about 0.1 bars. After the drying step, 1 g of bacitracin dissolved in 3 mL water was injected over the FDU-12 powder and allowed to be absorbed into the pores, at 1 bar. After this step, drying was achieved by applying a vacuum rate of 0.1 bar for about 30–60 min.

#### 3.2.6. Release of Bacitracin in Simulated Body Fluid (SBF)

The evaluation of the delivery of bacitracin was performed by placing ~100 mg of sample in 200 mL of simulated body fluid (SBF), keeping it at 37 °C, with stirring at 300 rpm. At each timepoint, 1 mL of liquid is collected and 1 mL of SBF is added back to compensate for the removed solution. The release process was performed in two types of SBF medium, with both having compositions similar to that of blood, and the first medium had a pH value of 7.4 [86] and the second one had a lower pH, at a value of 6.9, which mimics a blood infection [87]. When monitoring the bacitracin level, a high-pressure liquid chromatography separation method coupled with UV detection using a Diode Array Detector (HPLC-DAD) was used according to British Pharmacopeia [88]. The chromatographic conditions are summarized in Supplementary Table S1.

From a stock solution of bacitracin, 6 standardized solutions were obtained (in the same solvent as the release medium), corresponding to 1, 5, 25, 50, 100, 500 ppm. The standards are injected and the correlation between concentration and absorbance was determined based on linear equation of the curve from the graph: f(Area) = Concentration. The relative released bacitracin content is expressed as a percentage reported to the total content of bacitracin from the samples.

#### 3.2.7. Kinetic Models

Various kinetic models are available to address the issue of drug release modeling, with targeted applications for specific materials and shapes [89]. Among these models, three kinetic models were tested to fit the released bacitracin curve, i.e., Weibull model (denoted herein as W), Korsmeyer–Peppas model (denoted as K-P), and nonlinear regression (natural logarithm regression, denoted as LnR). Weibull model is defined according to Equation (4) [90]:

(4) $$M=M_{\infty }\left(1-e^{-\frac{t^{b}}{a}}\right)$$

where a—scale factor; b—shape factor; t—time; and M_∞_ is the maximum value of the released bacitracin mass, M, in mg. Parameters a and b are to be calculated during the fitting process. Other versions of the exponent were tested with poor results.

The Korsmeyer–Peppas model, given in Equation (5), has other two parameters that need to be obtained, i.e., k and n.

(5) $$M=M_{\infty }\cdot k\cdot t^{n}$$

As a peculiar feature, the K-P model could use only 60% of datasets in order to be effective [89] as compared to the W model. The effectiveness of using 60% of datasets as compared to 100% for the K-P model was also assessed.

Pairs of parameters (a, b) and (k, n), for the two models (W and K-P) were obtained by using the Generalized Reduced Gradient method; determination coefficients R^2^ between fitted and experimental results were also calculated and used to compare effectiveness. Natural logarithmic regression (LnR) was also used to evaluate whether a simpler, yet more effective equation could be identified.

#### 3.2.8. Characterization of Samples

Transmission Electron Microscopy (TEM) was used to provide information about the particle size and morphology of the material. The instrument used was a High-Resolution 80-200 TITAN THEMIS transmission microscope equipped with an Image Corrector 4 EDXS detectors in the column, manufactured by FEI (Hillsboro, OR, USA).

Scanning Electron Microscopy (SEM) was performed with a Inspect F50 microscope (FEI Company, Eindhoven, The Netherlands), and was equipped with a Schottky Field Emission source and a system capable of providing a vacuum in the sample chamber of 6 × 10^−4^ Pa. The acceleration voltage is controlled and varies from 200 V to 30 kV and the sample current is 200 nA. For analysis the samples were coated with a silver layer. For measurements the images were processed with ImageJ software, version 1.54g.

For confirmation of the specific cage structure of FDU-12, the X-ray diffraction (XRD) technique was used. The analysis was performed using CuKα PAN Analytical Empyrean powder diffractometer (PANalytical, Almelo, The Netherlands) in the Bragg–Brentano configuration. The measurement parameters included a 2θ angle range of 0.5–10, with a step size of 0.0256° and a time per step of one second.

The functional groups of samples were investigated with Fourier Transform-Infrared Spectroscopy (FT-IR) which was performed using a Nicolet iS50 FTIR with DTGS ATR detector (Thermo Fisher Scientific, Madison, WI, USA) by co-addition of 64 scans in the 4000–400 cm^−1^ interval, at a resolution of 8 cm^−1^. The validation of the presence of bacitracin in samples can be obtained through this type of analysis.

For determination of zeta potential, the analyses were performed in triplicate. First, the samples were dispersed in pure water using an ultrasonic bath, then they were placed inside the measurement cell of DelsaMax Pro equipment (Backman Coulter, Brea, CA, USA).

The efficiency of the functionalization process and of the loading process can be estimated according to data obtained from thermogravimetric analysis combined with differential scanning calorimetry (TGA-DSC). The instrument used was a Netzsch 449C STA Jupiter (Netzsch-Gerätebau GmbH, Selb, Germany) that worked from room temperature up to 900 °C with a heating rate of 10 °C/min in dry air (10 mL/min).

The textural properties were determined by N_2_ physisorption measurements at 77 K using a NOVA 2200e-Quantachrome Analyzer porosimeter (Anton Paar, Graz, Austria). Pore volume was estimated from the quantity of absorbed N_2_ at a relative pressure P/P_0_ = 0.9. Pore-size distribution was obtained from the isotherm desorption by applying the Barett–Joyner–Halenda (BJH) method. The specific surface area was determined using the Brunauer–Emmett–Teller (BET) equation.

#### 3.2.9. Evaluation of Antimicrobial Activity of Mesoporous Silica FDU-12 Loaded with Bacitracin Against *Staphylococcus* spp. Strains

The antimicrobial activity of mesoporous silica FDU-12 formulations loaded with bacitracin at different concentrations designated as B_0_ (bacitracin only), B_1_ (FDU-12 + bacitracin), B_4_ (FDU-12–NH_2_ + bacitracin), and BP (FDU-12–pNAM + bacitracin) was evaluated using both qualitative and quantitative antimicrobial assays.

##### Qualitative Screening of Mesoporous Silica FDU-12 Formulations Loaded with Bacitracin

The qualitative antimicrobial activity of mesoporous silica FDU-12 formulations loaded with bacitracin at different concentrations was evaluated. Antimicrobial screening was carried out against the reference strains *Staphylococcus aureus* ATCC 25923 and *Staphylococcus epidermidis* ATCC 12228, which were purchased from the American Type Culture Collection, (ATCC, Manassas, VA, USA), as well as eight clinical *S. aureus* isolates previously obtained and characterized from patients with hidradenitis suppurativa [91]. The evaluation was conducted using a modified disk diffusion method as previously described [92,93]. Standardized bacterial suspensions adjusted to a 0.5 McFarland standard (approximately 1.5 × 10^8^ CFU/mL) were uniformly inoculated onto the surfaces of Mueller Hinton (MH) agar plates. Subsequently, 10 µL aliquots of each bacitracin-loaded FDU-12 formulation (100 mg/mL) were spotted onto the inoculated plates. Following incubation at 37 °C for 24 h under aerobic conditions, antimicrobial efficiency was evaluated by measuring the diameters of the resulting inhibition zones. The inhibition was expressed in arbitrary activity units (AU) and defined as follows: AU 0 = no inhibition (0 mm); AU 1 = weak inhibition (<10 mm); AU 2 = moderate inhibition (10–20 mm); and AU 3 = strong inhibition (21–30 mm).

##### Quantitative Evaluation of Antimicrobial Activity of Mesoporous Silica FDU-12 Formulations Loaded with Bacitracin

The quantitative assessment of the antimicrobial activity of tested formulations (B_0_; B_1_; B_4_; and BP) was performed using the broth microdilution method in sterile 96-well microtiter plates. Serial twofold dilutions of each tested solution were prepared in Mueller–Hinton (MH) broth to yield final concentrations ranging from 50 to 0.09 mg/mL. A standardized bacterial suspension adjusted to 0.5 McFarland standard (1.5 × 10^8^ CFU/mL) was inoculated into each well to obtain a final inoculum representing 10% of the total well volume (100 µL). Plates were incubated at 37 °C for 24 h under aerobic conditions, and bacterial growth was measured spectrophotometrically at 620 nm (Thermo Scientific™ Multiskan™ GO). The minimum inhibitory concentration (MIC) was defined as the lowest concentration, showing complete growth inhibition. MIC values were obtained from triplicate assays, corrected for blanks, and expressed as mean ± standard deviation.

##### Evaluation of the Effect of Mesoporous Silica FDU-12 Formulations Loaded with Bacitracin on Microbial Adherence to an Inert Substratum

The effect of bacitracin-loaded FDU-12, FDU-12–NH_2_, and FDU-12–pNAM formulations on the adherence capacity of microbial strains to an inert substratum was evaluated using the crystal violet microtiter plate assay. The percentage of microbial adherence inhibition (%MAI) was calculated according to the following formula, Equation (6):

(6) $$\%\mathrm{MAI}\;=\;\frac{\left(As-Ablank\right)}{\left(Ac-Ablank\right)}\times 100$$

where As is the absorbance at 490 nm of treated samples, and Ac is the absorbance at 490 nm of the control [94].

##### Evaluation of the Influence of Bacitracin and Bacitracin–FDU-12 Derivatives on the Modulation of Soluble Virulence Factors

The effect of bacitracin and FDU-12, FDU-12–NH_2_, and FDU-12–pNAM loaded with bacitracin at different concentrations on the production of soluble virulence factors was investigated as previously described [95,96]. Microbial strains were cultivated in the presence of sub-inhibitory concentrations (MIC/2 and MIC/4) of each formulation on differential solid media supplemented with 5% sheep blood, 2.5% egg yolk, 1% starch, 1% Tween 80, and 15% soluble casein, to evaluate the modulation of virulence factor production, including hemolysins, lecithinase, amylase, lipase, and caseinase. The influence on virulence factor secretion was quantified according to the following relationship expressed by Equation (7):

(7) $$\mathrm{Inhibition}\;\%\;=\;\frac{\left(D2-C2\right)}{\left(D1-C1\right)}\times 100$$

where C1—diameter of culture spot untreated with B and FDU-12/FDU-12-NH_2_/FDU-12-pNAM loaded with B—control; D1—the diameter of the clear/precipitated zone around the culture spot untreated with B and FDU-12/FDU-12-NH_2_/FDU-12-pNAM loaded with B-control; C2—diameter of culture spot treated with B and FDU-12/FDU-12-NH_2_/FDU-12-pNAM loaded with B—sample; and D2—the diameter of the clear/precipitated zone around the culture spot treated with MIC/2 of B and FDU-12/FDU-12-NH_2_/FDU-12-pNAM loaded with B—sample.

##### Statistical Analysis

The results obtained from antimicrobial activity, anti-adherence, and virulence modulation assays were analyzed using GraphPad Prism version 10.0.0 for Windows (GraphPad Software, Boston, MA, USA, www.graphpad.com). Data are presented as mean ± standard deviation (SD). Statistical significance was assessed using two-way ANOVA followed by Dunnett’s multiple comparison test, with *p* < 0.05 considered statistically significant.

## 4. Conclusions

FDU-12 was prepared and modified with two functionalization agents, aminopropyl-triethoxysilane and poly(N-acryloyl-morpholine). Starting from these three supports, the loading of the bacitracin was performed and the release was evaluated considering two different simulated body fluids with same components but slightly different pH (pH = 7.4—standard and pH = 6.9—slightly acidic, conditions which can simulate infections) and found out that the surface modification can affect the release behavior, these systems being pH-sensitive.

Bacitracin exhibited strong antimicrobial activity against both reference and clinical *Staphylococcus* strains, while its incorporation into mesoporous silica FDU-12 derivatives maintained or slightly modulated this effect depending on the functionalization. The bacitracin-loaded FDU-12 formulations, particularly FDU-12–pNAM and FDU-12–NH_2_, demonstrated notable anti-adherence and biofilm inhibitory properties, though their efficacy was strain and concentration-dependent. Exposure to sub-inhibitory concentrations revealed variable modulation of soluble virulence factors, with some formulations reducing hemolysin, lipase, and amylase production, while others induced mild stimulatory effects in certain isolates. Overall, mesoporous silica carriers preserved bacitracin’s antimicrobial potency and enhanced its anti-biofilm potential, but sub-inhibitory exposure may differentially influence bacterial virulence expression, underscoring the importance of formulation selection and dosage optimization in therapeutic applications.

## Figures and Tables

**Table 1:** Textural characteristics of unloaded and loaded samples according to physisorption curves.

Sample	BET Surface Area (m^2^/g)	Total Pore Volume (cm^3^/g)
FDU-12	470.8	0.435
FDU-12 + Bacitracin	18.92	0.06829
FDU-12-NH_2_	129.3	0.3725
FDU-12-NH_2_ + Bacitracin	12.12	0.04312
FDU-12-pNAM	347.5	0.4499
FDU-12-pNAM + Bacitracin	7.13	0.01631

**Table 2:** Principal peaks of bacitracin.

Group	Bacitracin	FDU-12 + Bacitracin	FDU-12-NH_2_ + Bacitracin	FDU-12-pNAM + Bacitracin
N–H stretching (amide A, peptide NH)	3279	3285	3276	3285
Aliphatic, aromatic -CH stretching	2960	2963	2961	2963
Amide I band (C=O stretching of peptide bond)	1644	1652	1647	1647
Amide II band (N–H bending + C–N stretching)	1512	1526	1522	1526
Symmetric COO^−^ stretching	1386	1394	1394	1388
C-S stretching	699	700	698	699

**Table 3:** Thermal behavior of simple and functionalized FDU-12.

Sample	Mass Loss (%)				Residual Mass (%@900 °C)	Estimated Load (%)
	RT-120 °C	120–250 °C	250–440 °C	440–900 °C		
FDU-12	0.63	0.87	1.76	3.90	92.89	-
FDU-12-NH_2_	1.15	1.26	5.99	5.23	86.43	6.95
FDU-12-pNAM	1.26	6.52	2.48	5.00	84.72	8.80
FDU-12-Bacitracin	2.55	3.58	27.04	18.92	47.90	48.43
FDU-12-NH_2_-Bacitracin	2.66	3.94	30.11	20.88	42.36	50.99
FDU-12-pNAM-Bacitracin	2.94	5.99	25.65	19.63	45.81	45.93
Bacitracin	3.33	6.74	51.87	41.19	0	-

**Table 4:** Parameters and R^2^ values of the three models used. Weibull model used 100% of the datasets, Korsmeyer–Peppas model used 60%, while Logarithmic regression used both.

pH	Sample	W 100%			K-P 60%			LnR	
		a	b	R^2^	K	n	R^2^	R^2^ 100%	R^2^ 60%
7.4	FDU-12	30.440	0.719	0.98	0.080	0.500	0.98	0.85	0.85
	FDU-12-NH_2_	35.733	0.930	0.98	0.120	0.420	0.97	0.56	0.99
	FDU-12-pNAM	58.718	0.770	0.99	0.050	0.640	0.99	0.96	0.94
6.9	FDU-12	7.792	0.792	0.99	0.290	0.280	0.94	0.58	0.95
	FDU-12-NH_2_	86.920	0.903	0.99	0.033	0.688	0.97	0.91	0.85
	FDU-12-pNAM	17.244	0.643	0.99	0.100	0.500	0.95	0.89	0.94

**Table 5:** The inhibition zones diameters (mm) and the corresponding AU of the tested formulations against *S. aureus* ATCC 25923, *S. epidermidis* ATCC 12228, and clinical *S. aureus* isolates (AU—arbitrary units; ZD = inhibition zone diameter).

	Bacterial Strains																			
	*S. aureus* ATCC 25923		*S. epidermidis* ATCC 12228		*S. aureus* 30		*S. aureus* 32		*S. aureus* 39		*S. aureus* 44		*S. aureus* 59		*S. aureus* 60		*S. aureus* 68		*S. aureus* 100	
Tested Formulation	ZD	AU	ZD	AU	ZD	AU	ZD	AU	ZD	AU	ZD	AU	ZD	AU	ZD	AU	ZD	AU	ZD	AU
B0	19	2	25	3	21	3	21	3	22	3	22	3	23	3	20	2	21	3	21	3
B1	16	2	21	3	18	2	19	2	17	2	19	2	20	2	15	2	18	2	17	2
B4	11	2	17	2	13	2	14	2	15	2	14	2	13	2	10	1	14	2	14	2
BP	14	2	19	2	15	2	15	2	16	2	17	2	17	2	14	2	17	2	16	2

**Table 6:** Mean MIC values obtained from the quantitative evaluation of the antimicrobial activity of Bacitracin and Bacitracin–FDU-12 derivatives against reference strains and clinical isolates of *S. aureus*.

	B0		B1		BP		B4	
	MIC Mean (mg/mL)	St. Dev.	MIC Mean (mg/mL)	St. Dev.	MIC Mean (mg/mL)	St. Dev.	MIC Mean (mg/mL)	St. Dev.
*S. aureus* ATCC 25923	0.14	0.07	0.78	0	0.39	0	2.34	1.11
*S. epidermidis* ATCC 12228	0.09	0	0.19	0	0.29	0.14	0.78	0
60	0.39	0	2.34	1.11	1.56	0	1.17	0.55
59	0.39	0	1.56	0	1.56	0	3.13	0
44	0.39	0	0.59	0.28	1.56	0	4.69	2.21
100	0.39	0	0.78	0	1.56	0	7.81	6.63
32	0.39	0	1.17	0.55	1.56	0	7.81	6.63
39	0.19	0	0.59	0.28	0.78	0	3.13	0
30	0.39	0	1.56	0	1.56	0	3.13	0
68	1.56	0	6.25	0	3.125	0	9.38	4.42

**Table 7:** Percentage of microbial adherence inhibition (%MAI) of *S. aureus* ATCC 25923, *S. epidermidis* ATCC 12228, and clinical *S. aureus* isolates in the presence of Bacitracin and Bacitracin–FDU-12 derivatives at sub-inhibitory concentration (MIC/2).

	Strain Control		B0			B1			BP			B4		
	M	SD	M	SD	*p*	M	SD	*p*	M	SD	*p*	M	SD	*p*
60	100	50.04	50.13	27.70	0.392	16.21	7.75	0.055	48.27	7.58	0.360	32.18	37.92	0.1538
59	100	21.88	66.29	12.00	0.714	10.61	11.50	0.04	51.97	19.50	0.425	9.10	5.37	0.0332
44	100	27.06	34.98	32.10	0.181	69.75	28.93	0.782	14.31	14.11	0.048	64.55	47.3.8	0.6781
100	100	26.38	74.22	43.37	0.860	59.04	11.61	0.56	14.03	0.15	0.047	15.02	8.60	0.0507
32	100	34.95	28.05	35.47	0.120	197.19	64.17	0.021	53.79	12.60	0.459	32.01	36.87	0.1523
39	100	51.24	74.36	5.80	0.86	19.76	10.14	0.070	11.71	9.94	0.040	18.44	9.94	0.0643
30	100	46.22	35.96	1.140	0.191	45.20	55.97	0.311	157.11	43.75	0.277	122.35	22.73	0.9092
68	100	22.69	8.96	4.16	0.033	55.50	11.54	0.492	10.56	7.75	0.037	21.66	26.10	0.0798
*SA*	100	52.77	10.26	1.951	0.036	10.26	1.99	0.036	32.47	7.363	0.1565	93.72	131.46	0.9991
SE	100	36.08	66.67	1.79	0.721	21.31	22.38	0.078	28.69	24.465	0.1248	54.22	22.67	0.4676

M—mean value; SD—Standard Deviation; *p*—*p* value; SA—*S. aureus* ATCC 25923; SE—*S. epidermidis* ATCC 12228.

**Table 8:** Percentage of microbial adherence inhibition (%MAI) of *S. aureus* ATCC 25923, *S. epidermidis* ATCC 12228, and clinical *S. aureus* isolates in the presence of Bacitracin and Bacitracin–FDU-12 derivatives at sub-inhibitory concentration (MIC/4).

	Strain control		B0			B1			BP			B4		
	M	SD	M	SD	*p*	M	SD	*p*	M	SD	*p*	M	SD	*p*
60	100	50.04	37.07	0.33	0.429	33.23	44.35	0.376	74.85	5.61	0.945	18.07	12.37	0.211
59	100	21.88	43.48	14.50	0.52	66.11	2.75	0.860	55.50	3.00	0.711	29.61	8.12	0.331
44	100	27.06	38.45	9.81	0.448	71.70	4.50	0.919	89.77	31.28	0.99	80.38	61.94	0.977
100	100	26.38	70.58	8.97	0.909	75.26	13.82	0.948	61.43	6.32	0.799	39.66	3.90	0.466
32	100	34.95	121.45	4.67	0.968	268.15	197.20	0.002	20.79	5.13	0.236	62.05	42.94	0.808
39	100	51.24	0	0	0.093	37.47	12.42	0.434	40.98	57.13	0.485	17.27	19.87	0.204
30	100	46.22	146.06	29.83	0.687	96.03	48.02	>0.999	257.36	58.81	0.003	196.68	141.49	0.109
68	100	22.69	29.15	10.02	0.325	28.48	15.89	0.318	47.74	29.69	0.589	14.38	13.14	0.180
*SA*	100	52.77	0	0	0.093	25.57	4.55	0.285	26.49	31.40	0.295	61.41	29.45	0.799
*SE*	100	36.08	0	0	0.093	0	0	0.093	26.79	37.89	0.298	139.03	1.49	0.792

M—mean value; SD—Standard Deviation; *p*—*p* value; SA—*S. aureus* ATCC 25923; SE—*S. epidermidis* ATCC 12228.

**Table 9:** A summary table of the qualitative effects of Bacitracin and Bacitracin-FDU-12 formulations at sub-inhibitory concentration (MIC/2 and MIC/4) on *S. aureus* lecithinase activity.

Lecithinase		B0			B1			BP			B4		
Tested Strains	Subinhibitory Concentration	Positive Effect	Negative Effect	No Significant Effect	Positive Effect	Negative Effect	No Significant Effect	Positive Effect	Negative Effect	No Significant Effect	Positive Effect	Negative Effect	No Significant Effect
*S. aureus* ATCC 25923	MIC/2												
	MIC/4												
68	MIC/2												
	MIC/4												
32	MIC/2												
	MIC/4												
59	MIC/2												
	MIC/4												
60	MIC/2												
	MIC/4												
100	MIC/2												
	MIC/4												

Legend: gray—no statistically significant differences.

**Table 10:** A summary table of the qualitative effects of Bacitracin and Bacitracin-FDU-12 formulations at sub-inhibitory concentration (MIC/2 and MIC/4) on *S. aureus* caseinase activity.

Caseinase		B0			B1			BP			B4		
	Subinhibitory Concentration	Positive Effect	Negative Effect	No Significant Effect	Positive Effect	Negative Effect	No Significant Effect	Positive Effect	Negative Effect	No Significant Effect	Positive Effect	Negative Effect	No Significant Effect
SA	MIC/2												
	MIC/4												
30	MIC/2												
	MIC/4												
32	MIC/2												
	MIC/4												
39	MIC/2												
	MIC/4												
44	MIC/2												
	MIC/4												
59	MIC/2												
	MIC/4												
60	MIC/2												
	MIC/4												
100	MIC/2												
	MIC/4												
68	MIC/2												
	MIC/4												
SE	MIC/2												
	MIC/4												

SA—*S. aureus* ATCC 25923; SE—*S. epidermidis* ATCC 12228. Legend: gray—no statistically significant differences; green—stimulation; red—inhibition.

**Table 11:** A summary table of the qualitative effects of Bacitracin and Bacitracin-FDU-12 formulations at sub-inhibitory concentration (MIC/2 and MIC/4) on *S. aureus* amylase activity.

Amylase		B0			B1			BP			B4		
	Subinhibitory Concentration	Positive Effect	Negative Effect	No Significant Effect	Positive Effect	Negative Effect	No Significant Effect	Positive Effect	Negative Effect	No Significant Effect	Positive Effect	Negative Effect	No Significant Effect
SA	MIC/2												
	MIC/4												
30	MIC/2												
	MIC/4												
32	MIC/2												
	MIC/4												
39	MIC/2												
	MIC/4												
44	MIC/2												
	MIC/4												
59	MIC/2												
	MIC/4												
60	MIC/2												
	MIC/4												
100	MIC/2												
	MIC/4												
68	MIC/2												
	MIC/4												
SE	MIC/2												
	MIC/4												

SA—*S. aureus* ATCC 25923; SE—*S. epidermidis* ATCC 12228. Legend: gray—no statistically significant differences; green—stimulation; red—inhibition.

**Table 12:** A summary table of the qualitative effects of Bacitracin and Bacitracin-FDU-12 formulations at sub-inhibitory concentration (MIC/2 and MIC/4) on *S. aureus* lipase activity.

Lipase		B0			B1			BP			B4		
	Subinhibitory Concentration	Positive Effect	Negative Effect	No Significant Effect	Positive Effect	Negative Effect	No Significant Effect	Positive Effect	Negative Effect	No Significant Effect	Positive Effect	Negative Effect	No Significant Effect
SA	MIC/2												
	MIC/4												
32	MIC/2												
	MIC/4												
39	MIC/2												
	MIC/4												
44	MIC/2												
	MIC/4												
59	MIC/2												
	MIC/4												
60	MIC/2												
	MIC/4												
100	MIC/2												
	MIC/4												
68	MIC/2												
	MIC/4												
SE	MIC/2												
	MIC/4												

SA—*S. aureus* ATCC 25923; SE—*S. epidermidis* ATCC 12228. Legend: gray—no statistically significant differences; green—stimulation; red—inhibition.

**Table 13:** A summary table of the qualitative effects of Bacitracin and Bacitracin-FDU-12 formulations at sub-inhibitory concentration (MIC/2 and MIC/4) on *S. aureus* hemolysin activity.

Hemolysin		B0			B1			BP			B4		
	Subinhibitory Concentration	Positive Effect	Negative Effect	No Significant Effect	Positive Effect	Negative Effect	No Significant Effect	Positive Effect	Negative Effect	No Significant Effect	Positive Effect	Negative Effect	No Significant Effect
SA	MIC/2												
	MIC/4												
30	MIC/2												
	MIC/4												
32	MIC/2												
	MIC/4												
39	MIC/2												
	MIC/4												
44	MIC/2												
	MIC/4												
59	MIC/2												
	MIC/4												
60	MIC/2												
	MIC/4												
100	MIC/2												
	MIC/4												
68	MIC/2												
	MIC/4												

SA—*S. aureus* ATCC 25923. Legend: gray—no statistically significant differences; green—stimulation; red—inhibition.

## Data Availability

The research date will be available upon request.

## Supplementary Materials

The following supporting information can be downloaded at: https://www.mdpi.com/article/10.3390/molecules31020340/s1, Table S1: Chromatographic conditions of HPLC-DAD method.; Table S2: The influence of Bacitracin and Bacitracin–FDU-12 formulations at the corre-sponding subinhibitory MIC/2 and MIC/4 concentrations on lecithinase production in the selected *S. aureus* ATCC 25923 strain; Table S3: The influence of Bacitracin and Bacitracin–FDU-12 formula-tions at the corresponding subinhibitory MIC/2 and MIC/4 concentrations on lecithinase produc-tion in the selected clinical *S. aureus* 68 strain; Table S4: The influence of Bacitracin and Bacitracin–FDU-12 formulations at the corresponding subinhibitory MIC/2 and MIC/4 concentrations on le-cithinase production in the selected clinical *S. aureus* 32 strain; Table S5: The influence of Bacitracin and Bacitracin–FDU-12 formulations at the corresponding subinhibitory MIC/2 and MIC/4 con-centrations on lecithinase production in the selected clinical *S. aureus* 59, *S. aureus* 60, *S. aureus* 100 strain; Table S6: The influence of Bacitracin and Bacitracin–FDU-12 formulations at the corresponding subinhibitory MIC/2 and MIC/4 concentrations on caseinase production in the selected *S. aureus* strains; Table S7: The influence of Bacitracin and Bacitracin–FDU-12 formulations at the corresponding subinhibitory MIC/2 and MIC/4 concentrations on caseinase production in the selected *S. aureus* 68 strain; Table S8: The in-fluence of Bacitracin and Bacitracin–FDU-12 formulations at the corresponding subinhibitory MIC/2 and MIC/4 concentrations on caseinase production in the selected *S. epidermidis* ATCC 12228 strain; Table S9: The influence of Bacitracin and Bacitracin–FDU-12 formulations at the corresponding subinhibitory MIC/2 and MIC/4 concentrations on amylase production in the selected *S. aureus* 60, *S. aureus* 100 strain; Table S10: The influence of Bacitracin and Bacitracin–FDU-12 formulations at the corresponding subinhibitory MIC/2 and MIC/4 concentrations on amylase production in the selected *S. aureus* ATCC 25923, *S. epidermidis* ATCC 12228, *S. aureus* 30, *S. aureus* 32, *S. aureus* 39, *S. aureus* 44, *S. aureus* 59, *S. aureus* 60 strains; Table S11: The influence of Bacitracin and Bacitracin–FDU-12 formulations at the corresponding subinhibitory MIC/2 and MIC/4 concentrations on lipase production in the selected *S. aureus* ATCC 25923, *S. epidermidis* ATCC 12228, *S. aureus* 32, *S. aureus* 39, *S. aureus* 44, *S. aureus* 59, *S. aureus* 60, *S. aureus* 100 strains; Table S12: The influence of Bacitracin and Bacitracin–FDU-12 formulations at the corresponding subinhibitory MIC/2 and MIC/4 concentrations on lipase production in the selected *S. aureus* 68 strain; Table S13: The influence of Bacitracin and Bacitracin–FDU-12 formulations at the corresponding subinhibitory MIC/2 and MIC/4 concentrations on hemolysin production in the selected *S. aureus* ATCC 25923, *S. aureus* 30, *S. aureus* 32, *S. aureus* 39, *S. aureus* 44, *S. aureus* 59, *S. aureus* 60, *S. aureus* 68, *S. aureus* 100 strains.
