# Supplementary material for: Antimicrobial Functionalized Mesoporous Silica FDU-12 Loaded with Bacitracin
Source: Molecules. 2026 Jan 19;31(2):340. doi: 10.3390/molecules31020340 (PMC12844483; doi:10.3390/molecules31020340)
Supplement: Supplementary file 1 [file molecules-31-00340-s001.zip › molecules-4028201-supplementary update.pdf]

Supplementary Material

Antimicrobial functionalized mesoporous silica FDU-12 loaded with bacitracin

Dan Adrian Vasile [1,2,3], Ludmila Motelica [2,3,4], Luiza-Andreea Mîrț [1,2,3,5], Gabriel Vasilievici [5], Oana-Maria Memecică [6], Ovidiu Cristian Oprea [2,3,7,8], Adrian-Vasile Surdu [1,2,3,9], Roxana Doina Trușcă [2,3], Cristina Chircov [1,2,3], Bogdan Ștefan Vasile [2,3,4], Zeno Dorian Ghizdavet [1,2,3], Denisa Ficai [1,2,3,7,8], Ana-Maria Albu [6], Radu Pericleanu [10], Andreea Ștefania Dumbravă [10], Mara-Mădălina Mihai [11,12], Irina Gheorghe-Barbu [10,12] and Anton Ficai [1,2,3,8,*]

* Correspondence: anton.ficai@upb.ro

1. Supplementary Figures and Tables

1.1. Supplementary Tables

Supplementary Table S1. Chromatographic conditions of HPLC-DAD method.

**Characteristic**	**Condition**
Mobile Phase	Acetonitrile: Buffer solution pH=6: H_2O: Methanol 43: 100: 300: 557 (v/v/v/v)
Column	C18 150mm x 4.6 mm 5µm
Column Temperature	28 ^{o}C
Injection Volume	100 µL
Detector Wavelength	254 nm ± 2 nm

Supplementary Table S2. The influence of Bacitracin and Bacitracin–FDU-12 formulations at the corresponding subinhibitory MIC/2 and MIC/4 concentrations on lecithinase production in the selected *S. aureus* ATCC 25923 strain.

	Strain control			B0			B1			BP		
	mean	SD		mean	SD	p-value	mean	SD	p-value	mean	SD	p-value
***S. aureus* ATCC 25923**	100	21.65	**MIC/2**	75	0	0.5983	137.5	21.65	0.2708	137.5	21.65	0.2708
			**MIC/4**	87.5	21.65	0.9515	112.5	0	0.9515	125	21.65	0.5983

Supplementary Table S3. The influence of Bacitracin and Bacitracin–FDU-12 formulations at the corresponding subinhibitory MIC/2 and MIC/4 concentrations on lecithinase production in the selected clinical *S. aureus*$_{68}$ strain.

	Strain control			BP			B4		
	mean	SD		mean	SD	p-value	mean	SD	p-value
**68**	100	21.65	**MIC/2**	75	0	0.384	50	21.65	0.1069

	Strain control			B0			B1			BP			B4		
	mean	SD		mean	SD	p-value	mean	SD	p-value	mean	SD	p-value	mean	SD	p-value
**68**	100	21.65	**MIC/4**	75	0	0.3153	100	21.65	>0,9999	112.5	0	0.7755	112.5	0	0.7755

Supplementary Table S4. The influence of Bacitracin and Bacitracin–FDU-12 formulations at the corresponding subinhibitory MIC/2 and MIC/4 concentrations on lecithinase production in the selected clinical *S. aureus*$_{32}$ strain.

	Strain control			BP		
	mean	SD		mean	SD	p-value
**32**	100	21.65	**MIC/2**	87.5	21.65	0.7291
			**MIC/4**	75	0	0.384

Supplementary Table S5. The influence of Bacitracin and Bacitracin–FDU-12 formulations at the corresponding subinhibitory MIC/2 and MIC/4 concentrations on lecithinase production in the selected clinical *S. aureus*$_{59}$, *S. aureus*$_{60}$, *S. aureus*$_{100}$ strain.

	Strain control			B0			B1			BP			B4		
	mean	SD		mean	SD	p-value	mean	SD	p-value	mean	SD	p-value	mean	SD	p-value
**59**	100	24.74	**MIC/2**	85.71	0	0.945	128.57	0	0.4294	100	24.74	>0,9999	114.29	24.74	0.945
			**MIC/4**	85.71	0	0.945	100	24.74	>0,9999	114.29	24.74	0.945	128.57	0	0.4294
**60**	100	24.74	**MIC/2**	114.29	24.74	0.945	128.57	0	0.4294	114.29	24.74	0.945	128.57	0	0.4294
			**MIC/4**	114.29	24.74	0.945	128.57	0	0.4294	85.71	0	0.945	128.57	0	0.4294
**100**	100	0	**MIC/2**	111.11	19.25	0.986	77.78	19.25	0.6851	77.78	19.25	0.6851	111.11	19.25	0.986
			**MIC/4**	77.78	19.25	0.6851	111.11	19.25	0.986	100	0	>0,9999	100	0	>0,9999

Supplementary Table S6. The influence of Bacitracin and Bacitracin–FDU-12 formulations at the corresponding subinhibitory MIC/2 and MIC/4 concentrations on caseinase production in the selected *S. aureus* strains.

	Strain control			B0			B1			BP			B4		
	mean	SD		mean	SD		mean	SD		mean	SD		mean	SD	
**S. aureus ATCC 25923**	100	9.12	**MIC/2**	78.95	0	0.6626	63.16	0	0.1206	84.21	9.12	0.8816	63.16	0	0.1206
			**MIC/4**	73.68	9.12	0.424	63.16	15.79	0.1206	78.95	0	0.6626	68.42	9.12	0.2388
**30**	100	17.32	**MIC/2**	90	0	0.9896	80	17.32	0.7115	80	17.32	0.7115	120	0	0.7115
			**MIC/4**	120	0	0.7115	60	0	0.0762	90	0	0.9896	80	17.32	0.7115
**32**	100	17.32	**MIC/2**	90	0	0.9896	110	17.32	0.9896	110	17.32	0.9896	90	0	0.9896
			**MIC/4**	100	17.32	>0,9999	110	17.32	0.9896	80	17.32	0.7115	90	0	0.9896
**39**	100	24.74	**MIC/2**	157.14	24.74	**0.0036**	100	24.74	>0,9999	128.57	0	0.3362	100	24.74	>0,9999
			**MIC/4**	114.29	24.74	0.9252	100	24.74	>0,9999	157.14	24.74	**0.0036**	114.29	24.74	0.9252
**44**	100	12.37	**MIC/2**	78.57	12.37	0.645	64.29	21.43	0.1408	71.43	12.37	0.3362	57.14	12.37	**0.0489**
			**MIC/4**	64.29	0	0.1408	57.14	12.37	**0.0489**	57.14	12.37	**0.0489**	85.71	0	0.9252
**59**	100	21.65	**MIC/2**	125	21.65	0.4803	150	0	**0.0144**	150	37.5	**0.0144**	75	0	0.4803
			**MIC/4**	125	21.65	0.4803	137.5	21.65	0.1099	150	0	**0.0144**	87.5	21.65	0.9622
**60**	100	25	**MIC/2**	83.33	14.43	0.8513	66.67	14.43	0.1924	75	0	0.4803	116.67	14.43	0.8513
			**MIC/4**	100	0	>0,9999	91.67	14.43	0.9968	108.33	14.43	0.9968	100	25	>0,9999
**100**	100	21.65	**MIC/2**	137.5	21.65	0.1099	137.5	21.65	0.1099	112.5	0	0.9622	112.5	0	0.9622
			**MIC/4**	112.5	0	0.9622	150	0	**0.0144**	75	0	0.4803	100	21.65	>0,9999

Supplementary Table S7. The influence of Bacitracin and Bacitracin–FDU-12 formulations at the corresponding subinhibitory MIC/2 and MIC/4 concentrations on caseinase production in the selected *S. aureus*$_{68}$ strain.

	Strain control			BP			B4		
	mean	SD		mean	SD	p-value	mean	SD	p-value
**68**	100	24.74	**MIC/2**	114.29	24.74	0.8024	114.29	24.74	0.8024

	Strain control			B0			B1			BP			B4		
	mean	SD		mean	SD	p-value	mean	SD	p-value	mean	SD	p-value	mean	SD	p-value
**68**	100	24.74	**MIC/4**	142.86	24.74	0.278	114.29	24.74	0.9089	85.71	0	0.9089	100	24.74	>0,9999

Supplementary Table S8. The influence of Bacitracin and Bacitracin–FDU-12 formulations at the corresponding subinhibitory MIC/2 and MIC/4 concentrations on caseinase production in the selected *S. epidermidis* ATCC 12228 strain.

	Strain control			BP			B4		
	mean	SD		mean	SD	p-value	mean	SD	p-value
***S. epidermidis* ATCC 12228**	100	24.74	**MIC/2**	100	24.74	>0,9999	128.57	0	0.4558
			**MIC/4**	114.29	24.74	0.8641	128.57	0	0.4558

Supplementary Table S9. The influence of Bacitracin and Bacitracin–FDU-12 formulations at the corresponding subinhibitory MIC/2 and MIC/4 concentrations on amylase production in the selected *S. aureus*$_{60}$, *S. aureus*$_{100}$ strain.

	Strain control			B1			BP			B4		
	mean	SD		mean	SD	p-value	mean	SD	p-value	mean	SD	p-value
**68**	100	0	**MIC/2**	50	0	**<0,0001**	50	0	**<0,0001**	50	0	**<0,0001**
			**MIC/4**	50	0	**<0,0001**	50	0	**<0,0001**	50	0	**<0,0001**
**100**	100	0	**MIC/2**	50	0	**<0,0001**	50	0	**<0,0001**	50	0	**<0,0001**
			**MIC/4**	50	0	**<0,0001**	50	0	**<0,0001**	50	0	**<0,0001**

Supplementary Table S10. The influence of Bacitracin and Bacitracin–FDU-12 formulations at the corresponding subinhibitory MIC/2 and MIC/4 concentrations on amylase production in the selected *S. aureus* ATCC 25923, *S. epidermidis* ATCC 12228, *S. aureus*$_{30}$, *S. aureus*$_{32}$, *S. aureus*$_{39}$, *S. aureus*$_{44}$, *S. aureus*$_{59}$, *S. aureus*$_{60}$ strains.

	Strain control			B0			B1			BP			B4		
	mean	SD		mean	SD	p-value	mean	SD	p-value	mean	SD	p-value	mean	SD	p-value
***S. aureus* ATCC 25923**	100	0	**MIC/2**	100	0	>0,9999	100	0	>0,9999	100	0	>0,9999	100	0	>0,9999
			**MIC/4**	100	0	>0,9999	100	0	>0,9999	100	0	>0,9999	100	0	>0,9999
***S. epidermidis* ATCC 12228**	100	0	**MIC/2**	100	0	>0,9999	100	0	>0,9999	100	0	>0,9999	100	0	>0,9999
			**MIC/4**	100	0	>0,9999	100	0	>0,9999	100	0	>0,9999	100	0	>0,9999
**30**	100	34.64	**MIC/2**	60	0	**0.0229**	60	0	**0.0229**	120	0	0.5392	60	0	**0.0229**
			**MIC/4**	60	0	**0.0229**	60	0	**0.0229**	120	0	0.5392	60	0	**0.0229**
**32**	100	34.64	**MIC/2**	60	0	**0.0229**	60	0	**0.0229**	100	34.64	>0,9999	60	0	**0.0229**
			**MIC/4**	120	0	0.5392	80	34.64	0.5392	120	0	0.5392	60	0	**0.0229**
**39**	100	0	**MIC/2**	100	0	>0,9999	100	0	>0,9999	100	0	>0,9999	100	0	>0,9999
			**MIC/4**	100	0	>0,9999	166.67	57.74	**<0,0001**	100	0	>0,9999	100	0	>0,9999
**44**	100	0	**MIC/2**	100	0	>0,9999	100	0	>0,9999	100	0	>0,9999	100	0	>0,9999
			**MIC/4**	100	0	>0,9999	133.33	57.74	0.0824	100	0	>0,9999	100	0	>0,9999
**59**	100	0	**MIC/2**	50	0	**0.0024**	50	0	**0.0024**	50	0	**0.0024**	50	0	**0.0024**
			**MIC/4**	50	0	**0.0024**	50	0	**0.0024**	50	0	**0.0024**	50	0	**0.0024**
**60**	100	34.64	**MIC/2**	60	0	**0.0229**	60	0	**0.0229**	60	0	**0.0229**	60	0	**0.0229**
			**MIC/4**	60	0	**0.0229**	60	0	**0.0229**	60	0	**0.0229**	60	0	**0.0229**

Supplementary Table S11. The influence of Bacitracin and Bacitracin–FDU-12 formulations at the corresponding subinhibitory MIC/2 and MIC/4 concentrations on lipase production in the selected *S. aureus* ATCC 25923, *S. epidermidis* ATCC 12228*, S. aureus$_{32}$, S. aureus$_{39}$, S. aureus$_{44}$, S. aureus$_{59}$, S. aureus$_{60}$, S. aureus$_{100}$* strains.

	Strain control			B0			B1			BP			B4		
	mean	SD		mean	SD	p-value	mean	SD	p-value	mean	SD	p-value	mean	SD	p-value
***S. aureus* ATCC 25923**	100	0	**MIC/2**	58.33	14.43	0.0653	58.33	14.43	0.0653	83.33	14.43	0.8606	83.33	14.43	0.8606
			**MIC/4**	75	0	0.4975	66.67	14.43	0.2053	91.67	14.43	0.9971	83.33	14.43	0.8606
***S. epidermidis* ATCC 12228**	100	13.32	**MIC/2**	46.15	0	**0.0082**	69.23	0	0.2777	61.54	13.32	0.1043	23.08	0	**<0,0001**
			**MIC/4**	38.46	13.32	**0.0018**	61.54	13.32	0.1043	76.92	13.32	0.5843	46.15	0	**0.0082**
**32**	100	17.32	**MIC/2**	90	0	0.9906	110	17.32	0.9906	110	17.32	0.9906	90	0	0.9906
			**MIC/4**	100	17.32	>0,9999	110	17.32	0.9906	80	17.32	0.7257	90	0	0.9906
**39**	100	24.74	**MIC/2**	157.14	24.74	**0.0044**	100	24.74	>0,9999	128.57	0	0.3525	100	24.74	>0,9999
			**MIC/4**	114.29	24.74	0.9307	100	24.74	>0,9999	157.14	24.74	**0.0044**	114.29	24.74	0.9307
**44**	100	12.37	**MIC/2**	78.57	12.37	0.6606	64.29	21.43	0.1517	71.43	12.37	0.3525	57.14	12.37	0.0544
			**MIC/4**	64.29	0	0.1517	57.14	12.37	0.0544	57.14	12.37	0.0544	85.71	0	0.9307
**59**	100	21.65	**MIC/2**	125	21.65	0.4975	150	0	**0.0165**	150	37.5	**0.0165**	75	0	0.4975
			**MIC/4**	125	21.65	0.4975	137.5	21.65	0.1193	150	0	**0.0165**	87.5	21.65	0.9653
**60**	100	25	**MIC/2**	83.33	14.43	0.8606	66.67	14.43	0.2053	75	0	0.4975	116.67	14.43	0.8606
			**MIC/4**	100	0	>0,9999	91.67	14.43	0.9971	108.33	14.43	0.9971	100	25	>0,9999
**100**	100	21.65	**MIC/2**	137.5	21.65	0.1193	137.5	21.65	0.1193	112.5	0	0.9653	112.5	0	0.9653
			**MIC/4**	112.5	0	0.9653	150	0	**0.0165**	75	0	0.4975	100	21.65	>0,9999

Supplementary Table S12. The influence of Bacitracin and Bacitracin–FDU-12 formulations at the corresponding subinhibitory MIC/2 and MIC/4 concentrations on lipase production in the selected *S. aureus$_{68}$* strain.

	Strain control			BP			B4		
	mean	SD		mean	SD	p-value	mean	SD	p-value
**68**	100	24.74	**MIC/2**	114.29	24.74	0.8024	114.29	24.74	0.8024

	Strain control			B0			B1			BP			B4		
	mean	SD		mean	SD	p-value	mean	SD	p-value	mean	SD	p-value	mean	SD	p-value
**68**	100	24.74	**MIC/4**	142.86	24.74	0.278	114.29	24.74	0.9089	85.71	0	0.9089	100	24.74	>0,9999

Supplementary Table S13. The influence of Bacitracin and Bacitracin–FDU-12 formulations at the corresponding subinhibitory MIC/2 and MIC/4 concentrations on hemolysin production in the selected *S. aureus* ATCC 25923, *S. aureus*$_{30}$, *S. aureus*$_{32}$, *S. aureus*$_{39}$, *S. aureus*$_{44}$, *S. aureus*$_{59}$, *S. aureus*$_{60}$, *S. aureus*$_{68}$, *S. aureus*$_{100}$ strains.

	Strain control			B0			B1			BP			B4		
	mean	SD		mean	SD	p-value	mean	SD	p-value	mean	SD	p-value	mean	SD	p-value
***S. aureus* ATCC 25923**	100	24.74	**MIC/2**	85.71	0	0.5564	85.71	0	0.5564	85.71	42.86	0.5564	85.71	0	0.5564
			**MIC/4**	100	24.74	>0,9999	100	24.74	>0,9999	85.71	0	0.4119	71.43	24.74	**0.018**
**30**	100	13.32	**MIC/2**	92.31	0	0.9117	23.08	0	**<0,0001**	92.31	0	0.9117	115.38	0	0.4918
			**MIC/4**	92.31	0	0.8545	23.08	0	**<0,0001**	76.92	13.32	0.0743	92.31	0	0.8545
**32**	100	0	**MIC/2**	125	0	0.1131	25	0	**<0,0001**	91.67	14.43	0.8869	91.67	14.43	0.8868
			**MIC/4**	108.33	14.43	0.8174	25	0	**<0,0001**	100	0	>0,9999	108.33	14.43	0.8174
**39**	100	0	**MIC/2**	116.67	28.87	0.4204	116.67	28.87	0.4204	100	0	>0,9999	116.67	28.87	0.4204
			**MIC/4**	100	0	>0,9999	150	0	**<0,0001**	100	0	>0,9999	100	0	>0,9999
**44**	100	21.65	**MIC/2**	112.5	0	0.6645	100	21.65	>0,9999	0	0	**<0,0001**	125	21.65	0.1131
			**MIC/4**	112.5	0	0.5303	87.5	21.65	0.5303	0	0	**<0,0001**	112.5	21.65	0.5303
**59**	100	12.37	**MIC/2**	100	12.37	>0,9999	107.14	0	0.9305	92.86	12.37	0.9305	57.14	12.37	**0.0016**
			**MIC/4**	100	12.37	>0,9999	92.86	12.37	0.8833	100	12.37	>0,9999	50	12.37	**<0,0001**
**60**	100	12.37	**MIC/2**	157.14	12.37	**<0,0001**	128.57	21.43	0.0561	128.57	0	0.0561	64.29	0	**0.0109**
			**MIC/4**	142.86	12.37	**0.0002**	128.57	0	**0.018**	128.57	0	**0.018**	114.29	12.37	0.4119
**68**	100	0	**MIC/2**	0	0	**<0,0001**	58.33	14.43	**0.0023**	66.67	14.43	**0.0194**	66.67	14.43	**0.0194**
			**MIC/4**	0	0	**<0,0001**	91.67	14.43	0.8174	75	0	**0.0466**	75	0	**0.0466**
**100**	100	12.37	**MIC/2**	21.43	0	**<0,0001**	85.71	0	0.5564	78.57	12.37	0.2106	64.29	0	**0.0109**
			**MIC/4**	21.43	0	**<0,0001**	100	12.37	>0,9999	78.57	12.37	0.1082	71.43	12.37	**0.018**
